# Assessing earthworm exposure to a multi-pharmaceutical mixture in soil: unveiling insights through LC–MS and MALDI-MS analyses, and impact of biochar on pharmaceutical bioavailability

**DOI:** 10.1007/s11356-024-34389-1

**Published:** 2024-07-19

**Authors:** Jan Fučík, Rea Jarošová, Andreas Baumeister, Sascha Rexroth, Jitka Navrkalová, Marian Sedlář, Helena Zlámalová Gargošová, Ludmila Mravcová

**Affiliations:** 1 Institute of Chemistry and Technology of Environmental Protection, Faculty of Chemistry, Brno University of Technology, Purkyňova 118, 612 00 Brno, Czech Republic; 2 Veterinary Research Institute Brno, Hudcova 296/70, 621 00 Brno, Czech Republic; 3 Shimadzu Europa GmbH, Albert-Hahn-Straße 6, 472 69 Duisburg, Germany; 4 CEITEC Brno University of Technology, Purkyňova 656/123, 612 00 Brno, Czech Republic

**Keywords:** Pharmaceutical uptake, Soil pollution, Earthworms, Biochar, QuEChERS, Liquid chromatography, Mass spectrometry, MALDI-MS

## Abstract

In the European circular economy, agricultural practices introduce pharmaceutical (PhAC) residues into the terrestrial environment, posing a potential risk to earthworms. This study aimed to assess earthworm bioaccumulation factors (BAFs), the ecotoxicological effects of PhACs, the impact of biochar on PhAC bioavailability to earthworms, and their persistence in soil and investigate earthworm uptake mechanisms along with the spatial distribution of PhACs. Therefore, earthworms were exposed to contaminated soil for 21 days. The results revealed that BAFs ranged from 0.0216 to 0.329, with no significant ecotoxicological effects on earthworm weight or mortality (*p* > 0.05). Biochar significantly influenced the uptake of 14 PhACs on the first day (*p* < 0.05), with diminishing effects over time, and affected significantly the soil-degradation kinetics of 16 PhACs. Moreover, MALDI-MS analysis revealed that PhAC uptake occurs through both the dermal and oral pathways, as pharmaceuticals were distributed throughout the entire earthworm tissue without specific localization. In conclusion, this study suggests ineffective PhAC accumulation in earthworms, highlights the influence of biochar on PhAC degradation rates in soil, and suggests that uptake can occur through both earthworm skin and oral ingestion.

**Graphical Abstract:**

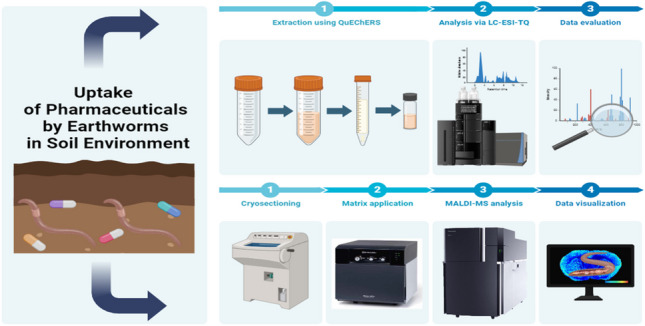

**Supplementary Information:**

The online version contains supplementary material available at 10.1007/s11356-024-34389-1.

## Introduction

Pharmaceuticals (PhACs) play a crucial role in disease management across human populations, animal husbandry, and aquaculture. Upon administration, pharmaceuticals, including antibiotics, are excreted via urine or feces, leading to the presence of pharmaceutical residues in treated wastewater, animal manure, and biosolids (Obimakinde et al. 2017; Valdez-Carrillo et al. 2020). In the context of the European circular economy, the application of these amendments is advocated to address challenges such as dwindling water resources, shortages of inorganic fertilizers, and the imperative to minimize waste while maximizing resource utilization in agricultural practices. However, within this circular economy framework, residues of emerging organic micropollutants, including PhACs, are dispersed into the terrestrial environment (Ammar et al. 2020; Wu et al. 2021; Kesari et al. 2021; Khare and Shikha 2023).

In contrast to their well-documented effects in aquatic environments, the impact of PhACs in soil environments remains inadequately characterized and understood (Gworek et al. 2021). The presence of these residues can negatively affect soil microbiota, which are essential for nutrient cycling, soil structure maintenance, plant disease suppression, and overall plant and soil organism health (Gallego and Martin-Laurent 2020; Sauvêtre et al. 2020). Even at trace concentrations, PhACs create an environment conducive to the rise of antimicrobial resistance (AMR) and further selective pressure toward resistant bacteria (Hashmi et al. 2017; Gallego and Martin-Laurent 2020). Statistical analyses conducted by study (Murray et al. 2022) have revealed that AMR is associated with up to 1.17 million deaths globally, with an additional 2.62 to 4.78 million deaths indirectly linked to AMR-related factors. Moreover, these drug residues can induce phytotoxicity, inhibit seed germination, alter plant morphology, and inhibit biomass growth (Hillis et al. 2011; Michelini et al. 2013). Additionally, pharmaceutical residues can be taken up by agricultural crops, leading to contamination of the food chain and ultimately reaching the original sources of environmental contamination, both humans and animals (Carter et al. 2014a, b). Furthermore, these residues can also negatively impact soil organisms, specifically *Eisenia fetida* (Carter et al. 2014a).

In the case of *E. fetida*, PhAC residues can trigger weight changes, elevate mortality rates, disrupt reproduction rates, and alter various aspects of earthworm behavior, such as feeding activity and burrowing behavior. Additionally, these residues can affect enzyme activity and lead to DNA alterations, ultimately impacting the overall health and population dynamics of this soil invertebrate species (Dong et al. 2012; Li et al. 2016). Moreover, similar to plants, the uptake and metabolization of these parent drugs can occur in earthworms, subsequently causing secondary contamination of earthworm predators and potential biomagnification (Carter et al. 2014a). Earthworms are also indispensable for soil quality because they decompose organic materials such as dead plant matter and animal residues, thereby accelerating the decomposition process. By shredding and consuming organic material, earthworms make nutrients more readily available for plant uptake, enriching the soil with humus, a dark, organic-rich substance that enhances soil fertility and moisture retention. This organic matter decomposition process rejuvenates the soil, replenishing essential nutrients and contributing to overall soil health and productivity (Garg et al. 2019). Therefore, earthworms are often utilized as sensitive indicators of soil quality, and a variety of biomarkers have been employed to assess the toxicological effects of organic contaminants (Li et al. 2016).

Currently, there is still a lack of a multiresidual extraction method for the quantification of PhACs in earthworm tissue. However, this research gap has been addressed in recent studies (Bergé and Vulliet 2015; Montemurro et al. 2021; Mravcová et al. 2024), where innovative methodologies such as QuEChERS or solid-phase extraction (SPE) have been developed. These methods enable the quantification of a broad spectrum of PhACs, exceeding 30 different compounds, within earthworm tissue. According to available scientific literature, PhAC concentrations both within soil and earthworm tissue typically ranged from ng∙g^−1^ to µg∙g^−1^ (Kinney et al. 2008; Carter et al. 2014a; Pan and Chu 2017; Cycoń et al. 2019). Furthermore, certain studies (Wen et al. 2011; Carter et al. 2014a; Chen et al. 2020) have investigated both the uptake and elimination phases of individual antibiotics, such as triclosan (Chen et al. 2020), fluoxetine and carbamazepine (Carter et al. 2014a), and ciprofloxacin (Wen et al. 2011). However, only a few studies (Bao et al. 2018; Li et al. 2016) have analyzed both concentrations within earthworm tissue and soil samples. Moreover, if bioaccumulation factors (BAFs) were calculated, they were often derived from one-time sampling at a single soil concentration, potentially leading to inaccuracies in reported BAFs. Furthermore, BAFs represent valuable information for decision-making regarding the application of contaminated amendments to agricultural fields. Elevated BAF values can serve as warning signs that the application of specific substances may result in accumulation in soil organisms, including *E. fetida*, as well as in plants and other soil invertebrates. However, a comprehensive assessment must also consider factors such as substance toxicity, degradation properties in soil, and the potential for AMR emergence. Despite the recognition of PhACs in the environment as a pressing issue by organizations such as the World Health Organization (WHO), the United Nations Environment Programme (UNEP), and the Organisation for Economic Co-operation and Development (OECD), no regulatory policies have been implemented to this date (Bartrons and Peñuelas 2017; Gros et al. 2019).

Furthermore, studies (Lin et al. 2021; Zhang et al. 2022) have successfully investigated the potential of earthworms in soil vermi-remediation, reporting successful remediation of tetracycline (Lin et al. 2021), sulfamethoxazole, and their antimicrobial resistance genes (ARGs) (Zhang et al. 2022), and heavy metals through the tandem use of biochar and earthworms (Khan et al. 2019). However, there is still a lack of studies investigating the effect of biochar on earthworm uptake and the degradation rate of PhACs in the soil environment, as has been conducted in studies on plant uptake (Li et al. 2020; Williams et al. 2015). Moreover, biochar offers a plethora of soil-enhancing attributes, encompassing heightened water retention, increased surface area, enhanced nutrient exchange, carbon sequestration, advancement of soil and plant health, augmentation of microbial communities, and mitigation of heavy metal and organic contaminant absorption (Majumder et al. 2023; Chen et al. 2023). These qualities position biochar as a valuable asset within the circular economy (Hu et al. 2021).

Additionally, various studies have examined the spatial distribution of diverse organic micropollutants across different tissues using mass spectrometry imaging (MSI). For instance, research has investigated the presence of herbicides in maize (Kubicki et al. 2022), insecticides in diamondback moth (Wan et al. 2022), phthalate esters in carrots (Xiang et al. 2022), fungicides in wheat leaves (Annangudi et al. 2015), iodosulfuron and quinestrol in *Salix alba* leaves (Villette et al. 2019), and beta-blockers in fathead minnows (Davis et al. 2020). Despite these investigations, there remains a notable gap in the literature concerning the examination of veterinary antibiotic distribution within earthworm tissues utilizing MSI techniques. Considering the vital role of earthworms in soil ecosystems and their potential as vectors for the transport and bioaccumulation of environmental contaminants like veterinary antibiotics, investigating the spatial distribution of veterinary antibiotics within earthworm tissues could provide valuable insights into the mechanisms of uptake, tissue localization, biotransformation pathways, and ecological implications (Annangudi et al. 2015; Davis et al. 2020; Kubicki et al. 2022; Villette et al. 2019; Wan et al. 2022; Xiang et al. 2022).

To address the existing research gaps, earthworms were exposed to a mixture of 21 PhACs at soil concentrations ranging from 0 to 20000 ng∙g^−1^ for a period of 21 days. The selected PhACs represent various pharmaceutical groups, including beta-blocking agents, tetracyclines, sulfonamides, macrolides, and fluoroquinolones. The selection of pharmaceutical substances in the study followed a prioritization process, considering factors such as high prevalence, potential environmental impact (e.g., if they belong to critically important antimicrobials), reported sales within the EU (European Medicines Agency 2021), and findings from other environmental and monitoring studies (Kinney et al. 2008; Carter et al. 2014a; Jesus Gaffney et al. 2017; Pan and Chu 2017; Souza et al. 2018; Gros et al. 2019; Cycoń et al. 2019; Kodešová et al. 2019; Ajibola et al. 2020; Riva et al. 2021). Consequently, BAFs for these pharmaceuticals were calculated using a novel approach of time-weighted average (TWA) soil concentration, contrary to published studies that either used only the initial soil concentration (Li et al. 2016; Bao et al. 2018; Yang et al. 2023) or soil concentration at sampling time to estimate BAFs  (Schmidt et al. 2022), which may lead to underestimation or overestimation of BAF values. Furthermore, earthworms were exposed to the PhAC mixture at a soil concentration of 10000 ng∙g^−1^, both in biochar-amended and non-amended soil. Soil and earthworm samples were obtained on days 1, 3, 7, 14, and 21. Consequently, the soil-degradation kinetics of individual PhACs were evaluated and followed first-order kinetics. The impact of biochar on bioavailability to earthworms and on the degradation of PhACs in soil was evaluated using statistical analyses. Moreover, ecotoxicological results on earthworm relative weight changes were obtained, statistically evaluated, and discussed in comparison with available studies. Lastly, to fill another research gap, the spatial distribution of selected veterinary antibiotics within earthworm tissue was investigated using MSI, specifically MALDI-MS imaging, with the aim of comprehensively characterizing their uptake and distribution patterns.

## Materials and methods

### Materials, chemicals, and standards

An overview of the chemicals, standards, and materials used in this study is provided in the supporting information (Appendix 1). These are appropriately divided into sections: Chemicals and materials for sample extraction and analysis, chemicals and consumables for cryosectioning and MALDI-Imaging-HRMS, and pharmaceutical standards. Additionally, a summary of the physicochemical properties of the pharmaceuticals used in this study is presented in Table S1.

### Earthworm exposure experiment, concentration range

Earthworms (*E. fetida*) were purchased from a local fish store (ProRyby, Czech Republic). The uptake experiment was performed according to the guidelines of the Organisation for Economic Cooperation and Development (OECD) Test No. 317 (OECD Author collective 2010) and similarly to studies (Carter et al. 2014a; Mravcová et al. 2024). Before the start of the experiment, earthworms with an average weight of 400 mg (recommended weight by OECD is 250–600 mg) were acclimatized for 3 days in non-contaminated soil (physicochemical properties of soil in Table S2). The acclimatization phase was followed by the depuration phase for 1 day (on filtration paper in dark). The soil was spiked with a mixture of 21 pharmaceuticals at concentrations of 100, 500, 750, 1000, 2000, 5000, 10000, and 20000 ng∙g^−1^ dw of soil. Subsequently, 50 ± 0.1 g of dry soil was weighed into each 100 mL beaker, and one earthworm per beaker was transferred into the soil. A total of 9 replicates were conducted for each concentration, including the control experiment. The beakers were covered with perforated food plastic wrap. The soil humidity was adjusted to 40% maximum water holding capacity (WHC_max_; checked and adjusted every third day), and the temperature during the experiment was maintained at 20 ± 1 °C for 24 h under light. Earthworms were fed with flakes on the first day of the experiment, and then every third day of the experiment. Earthworms were sampled for the depuration phase after 21 days of exposure, and the depuration phase lasted 1 day. After depuration, the earthworms were lyophilized and extracted using the QuEChERS method.

### Earthworm exposure experiment, impact of biochar

The exposure experiment was meticulously conducted following a standardized methodology outlined in the abovementioned protocol. However, a pivotal modification was introduced whereby the soil was deliberately contaminated with a mixture of 21 pharmaceuticals at a concentration of 10000 ng∙g^−1^, encompassing both non-amended and biochar-amended soil at a concentration of 2% w/w soil. Detailed physicochemical properties of biochar are provided in Table S3 originating from the study (Holatko et al. 2023). Sampling of both earthworms and soil occurred at intervals of 1, 3, 7, 14, and 21 days after-exposure.

### Earthworm exposure experiment, samples for mass spectrometry imagining

The exposure experiment was meticulously executed with uniform methodology, adhering to the aforementioned protocol. However, a critical deviation was introduced wherein the soil was enriched with a mixture of only 3 veterinary antibiotics (enrofloxacin, tetracycline, erythromycin) at a concentration of 10000 ng∙g^−1^ dry weight of soil. Subsequent to exposure periods of 1, 3, and 5 days to the PhAC mixture, earthworm samples were collected. Following depuration, the earthworms were subjected to sample preparation for MALDI analysis.

### Extraction of pharmaceuticals from earthworms and soil

Both earthworm and soil samples were extracted using our own previously validated and published methods (Mravcová et al. 2024), described in detail in the Supporting Information, appendix 2. For earthworms, the QuEChERS extraction technique was utilized, while soil samples were subjected to ultrasound-assisted extraction, followed by solid-phase extraction. Consequently, these extracts were analyzed by LC–MS/MS method (Mravcová et al. 2024), described in the Supporting Information, appendix 3.

### Sample preparation for MALDI-imaging-HRMS

Cryohistology was performed with small modifications as described previously (Jarošová et al. 2021). Earthworms were euthanized by freezing (− 20 °C), embedded into OCT compound, and cut to a thickness of 10–15 µm on the cryostat (CM1900, Leica Microsystems GmbH, Germany) at − 20 °C. The cuts were placed on ITO-coated slides, and the sections were allowed to dry at room temperature and processed according to the Cryostat Sectioning manual (Bruker 2018). DHB (30 mg∙mL^−1^ in MeOH:H_2_O 70:30 + 0.25% TFA was sprayed on the sample using iMLayer AERO (Shimadzu, Japan). Matrix application was performed under the following conditions: stage speed 40 mm∙s^−1^; drying time: 30 s, number of layers: 8; wash frequency (layer/wash): 8; nozzle distance: 5 cm; and scan pitch: 1 mm.

#### MALDI*-*imaging*-*HRMS

Optical images of earthworms were obtained using an optical microscope equipped within iMScope™ QT (Shimadzu, Japan) before matrix application. MSI measurements were performed using the same instrument fitted with a laser unit (Nd:YAG laser, 355 nm) operating under atmospheric pressure. The following laser firing parameters were used for the analysis: number of laser shots: 80; repetition rate: 2000 Hz; laser diameter: 5 µm; pitch size: 20.0 µm × 20.0 µm; and laser intensity: 77.6. The iMScope™ QT was coupled to an LCMS-9030 (Shimadzu, Japan) that operated in positive ion mode scanning m/z in the range of 100–1000. Before each MALDI-MS analysis, the mass accuracy was calibrated to the matrix cluster ions in a scanning range of up to 1000. For data processing, IMAGEREVEAL MS software (Shimadzu, Japan) and MSiReader v2.7 (MSI Software Solutions, LLC, Raleigh, NC) were used.

## Results and discussion

### Earthworm exposure to different concentrations of pharmaceuticals

Earthworms were subjected to a mixture of 21 PhACs, including Beta-blocking agents, Fluoroquinolones, Macrolides, Tetracyclines, and Sulfonamides, in a terrestrial environment under controlled laboratory conditions for 21 days. To comprehensively assess BAFs, the soil was contaminated at concentrations ranging from 0 to 20000 ng∙g^−1^. These soil concentrations spanned from environmentally relevant levels (~ units to hundreds of ng∙g^−1^) as reported in studies (Monteiro and Boxall 2010; Gao et al. 2015; Zhang et al. 2017; Zhao et al. 2018; Gworek et al. 2021), to higher concentrations (thousands to tens of thousands of ng∙g^−1^) as reported in studies (Wei et al. 2016; Guo et al. 2022), which can be found in manure-amended soil. Animal manure can contain PhAC concentrations as high as hundreds to thousands of mg∙kg^−1^, if not treated appropriately before incorporation into the soil (Zhao et al. 2010). BAFs in earthworms or plants are commonly calculated using Eq. 1. Some studies (Li et al. 2016; Bao et al. 2018; Yang et al. 2023) utilized the initial concentrations of exposure, leading to an underestimation of BAFs, whereas study (Schmidt et al. 2022) solely used concentrations at the time of sampling, potentially resulting in overestimation of BAFs that do not reflect real conditions. This discrepancy in the BAF estimation is attributed to the degradation of PhACs in the soil environment. Degradation rates (k [d^−1^], in Table 1) were determined by linear regression using Eq. 2 for all individual substances in the terrestrial environment. Consistent with previous studies (Xu et al. 2009; Zhang et al. 2013; Sidhu et al. 2019), degradation followed first-order kinetics, indicating that the initial concentration does not influence the degradation rate.

1 $$\text{BAF}=\frac{{c}_{\text{earthworm}}}{{c}_{\text{soil}}}$$

where *c*_earthworm_ [ng∙g^−1^] represents the concentration in earthworms at the end of the experiment, while *c*_soil_ [ng∙g^−1^] indicates the soil concentration, which could be the initial concentration, the concentration at the experiment's conclusion, or the time-weighted average concentration.

2 $$\text{ln}\left(\frac{{\text{c}}_0}{{\text{c}}_\text{end}}\right)=\text{k}{\cdot\text{t}}_\text{end}$$

where *c*_0_ [ng∙g^−1^] represents the initial soil concentration, *c*_end_ [ng∙g^−1^] is the concentration at the end of the experiment, k [d^−1^] indicates the degradation rate kinetics, and *t*_end_ [day] stands for the experiment’s duration.

3 $$c_{\text{soil},\;\mathrm{avg}.}=\frac1{{\text{t}}_\text{end}}\int_{t_0}^{{\text{t}}_\text{end}}{\text{c}}_0\cdot\text{e}^{-\text{kt}}\text{dt}$$

where *c*_soil,avg._ [ng∙g^**−**1^] denotes the time-weighted average soil concentration throughout the experiment, *t*_end_ [day] stands for the experiment’s duration, *c*_0_ [ng∙g^−1^] represents the initial soil concentration, and k [d^−1^] stands for the degradation rate kinetics.

**Table 1:** Results of PhAC uptake by earthworms**.** Bioaccumulation factors (BAFs) and standard error (SE) for individual pharmaceuticals after 21 days and degradation rate kinetics (k) and standard error (SE) of pharmaceuticals in biochar-amended and non-amended soil; *R*^2^—coefficient of determination; D_21D_—degradation after 21 days

Pharmaceutical group	Pharmaceutical name	Bioaccumulation factor in biochar non-amended soil				Non-amended soil					Biochar-amended soil			
		BAF [-]	SE (BAF) [-]	*R*^2^ [-]	Linearity range [ng∙g^−1^]	k [d^−1^]	SE (k) [d^−1^]	*R*^2^ [-]	D_21D_ [%]	k [d^−1^]		SE (k) [d^−1^]	R^2^ [-]	D_21D_ [%]
Beta Blockers	Acebutolol	0.193	0.016	0.969	Up to 2000	0.032	0.004	0.9626	48	0.032		0.007	0.8779	49
Macrolides	Azithromycin	0.027	0.003	0.941	Up to 10000	0.021	0.003	0.9291	30	0.032		0.006	0.8709	40
Fluoroquinolones	Ciprofloxacin	0.0216	0.0005	0.998	Up to 20000	0.056	0.006	0.9494	65	0.043		0.009	0.8625	49
Macrolides	Clarithromycin	0.057	0.003	0.992	Up to 10000	0.0336	0.0007	0.9992	27	0.020831		0.000024	1.0000	35
Fluoroquinolones	Enrofloxacin	0.0402	0.0018	0.992	Up to 10000	0.06	0.01	0.9298	69	0.035		0.009	0.8440	45
Macrolides	Erythromycin	0.317	0.010	0.997	Up to 20000	0.033	0.002	0.9838	24	0.00592		0.00020	0,6336	12
Tetracyclines	Chlortetracycline	0.0568	0.0012	0.997	Up to 20000	0.076	0.006	0.9807	76	0.063		0.010	0.9135	67
Fluoroquinolones	Moxifloxacin	0.063	0.011	0.995	Up to 5000	0.069	0.007	0.9543	72	0.068		0.018	0.8185	47
Beta Blockers	Nadolol	0.154	0.004	0.996	Up to 20000	0.131	0.014	0.9460	91	0.098		0.009	0.9599	85
Fluoroquinolones	Ofloxacin	0.0309	0.0012	0.993	Up to 20000	0.044	0.005	0.9504	56	0.033		0.006	0.9061	42
Tetracyclines	Oxytetracycline	0.0253	0.0020	0.975	Up to 10000	0.0446	0.0016	0.9963	59	0.024		0.004	0.9222	43
Macrolides	Roxithromycin	0.111	0.007	0.973	Up to 10000	0.0413	0.0022	0.9916	26	0.0120		0.0019	0.9289	24
Sulfonamides	Sulfacetamide	0.163	0.005	0.995	Up to 20000	0.170	0.015	0.9643	96	0.157		0.006	0.9932	96
Sulfonamides	Sulfadimethoxine	0.0616	0.0024	0.992	Up to 20000	0.114	0.015	0.9359	89	0.0153		0.0010	0.9923	26
Sulfonamides	Sulfamethazine	0.0562	0.0023	0.987	Up to 20000	0.045	0.008	0.9307	66	0.063		0.006	0.9609	76
Sulfonamides	Sulfamethoxazole	0.329	0.012	0.991	Up to 20000	0.091	0.005	0.9882	83	0.075		0.005	0.9835	77
Sulfonamides	Sulfamethoxypyridazine	0.163	0.011	0.977	Up to 10000	0.045	0.005	0.9400	63	0.0645		0.0022	0.9955	74
Sulfonamides	Sulfapyridine	0.0875	0.0013	0.999	Up to 20000	0.082	0.004	0.9886	80	0.050		0.005	0.9660	67
Sulfonamides	Sulfathiazole	0.092	0.003	0.996	Up to 20000	0.121	0.013	0.9543	89	0.100		0.010	0.9587	84
Tetracyclines	Tetracycline	0.0467	0.0014	0.997	Up to 20000	0.088	0.010	0.9473	82	0.049		0.005	0.9668	60
Sulfonamides	Trimethoprim	0.052	0.005	0.961	Up to 20000	0.045	0.010	0.8629	52	0.030		0.005	0.9511	41

Due to the degradation of PhACs, earthworms are not consistently exposed to the same concentrations the entire experiment. Therefore, the time-weighted average (TWA) soil concentrations were calculated using Eq. 3, and subsequently, these concentrations were input into Eq. 1. Therefore, BAFs were determined from concentration series (0–20000 ng∙g^−1^) as the slope of the linear equation *y* = ax (where *y* is the concentration of pharmaceutical within the earthworm tissue, *x* is the TWA soil concentration, and *a* (slope) represents BAF). TWA concentrations are commonly utilized in the environmental analysis of PhACs, particularly in the case of passive samplers (Togola and Budzinski 2007; Mazzella et al. 2008). Furthermore, studies (Ma et al. 2014; Zhu et al. 2023) suggested that employing TWA concentration would be more suitable than using one-time sampling for the determination of BAFs. However, to the best of our knowledge, available studies have not yet utilized the TWA concentration for estimating BAFs. Overall, this approach offers a more nuanced and representative measure, especially in situations where concentrations change over time. By integrating time into the averaging process, more emphasis is placed on periods when the variable exhibits higher values and longer durations, resulting in a more accurate average. This is because both the initial concentration and the concentration at sampling time contribute to the BAF value. The BAFs for individual pharmaceuticals, along with the coefficients of determination and linearity ranges, can be found in Table 1.

When the BAF value > 1, effective bioaccumulation occurs, indicating that the substance is accumulating in the organism’s tissue. Conversely, when the BAF value < 1, uptake occurs, but bioaccumulation is not effective as the substance is not accumulating in the organism’s tissue at a significant rate. The bioaccumulation of these pharmaceuticals is well described by linear models, with coefficients of determination (*R*^2^) exceeding 0.96, consistent with the findings of the study (Sidhu et al. 2019). The high linearity indicates that the concentrations within earthworm tissue are proportional to the soil concentration, as reported in the study (Sidhu et al. 2019). Additionally, some PhACs exhibited linearity only within specific concentration ranges. This phenomenon could be attributed to various factors, including higher elimination rates due to elevated concentrations exceeding certain thresholds, limitations of the linear model, or experimental artifacts such as inaccuracies in measuring high concentrations, uneven distribution of pharmaceuticals in soil, or variability in earthworm size, health, or age, all of which could influence their bioaccumulation capacity.

Within our study, the obtained BAF values after 21 days of exposure were as follows: 0.154 to 0.193 for Beta-Blocking Agents, 0.027 to 0.317 for Macrolides, 0.0216 to 0.063 for Fluoroquinolones, 0.0253 to 0.0568 for Tetracyclines, and 0.052 to 0.329 for Sulfonamides. These findings indicate that no effective bioaccumulation was observed for earthworms, aligning with previous studies (Li et al. 2016; Bao et al. 2018). For instance, in the study by Bao et al. (2018), a BAF of 0.034 for oxytetracycline was reported after 28 days of exposure, while Li et al.’s (2016) study found BAFs for enrofloxacin in the range of 0.08 to 0.18 after 8 weeks of exposure. In contrast, an article by Sidhu et al. (2019) reported BAFs > 1 for ciprofloxacin (3.7057) and azithromycin (7.013) after 28 days of exposure. The majority of studies estimated BAF for a single compound (Li et al. 2016; Bao et al. 2018) or only for a few PhACs (< 2) (Sidhu et al. 2019), whereas our study estimated BAFs for 21 PhACs. Consequently, to assess which properties affect bioaccumulation, BAF values were plotted against their Mw, logP, and pKa values. However, no significant linear or non-linear correlations were found, in contrast to the findings of a study (Hu et al. 2023), where logBAF showed a significant negative correlation with logKow. Nevertheless, as suggested by the study (González-Alcaraz et al. 2020), the BAF is dependent on the partitioning coefficient (Kp), as it defines the dynamic process of chemical sorption and desorption to soil particles, leading to the presence of contaminants in pore water. This process is primarily driven by soil properties (e.g., organic carbon content, pH, Ca^2+^, and Mg^2+^ concentrations) and chemical properties (e.g., Kow). Additionally, the lipid content of the organism, in this case, the earthworm, can also influence the uptake rate and may vary between different earthworm species (5.1% wet weight in the case of *E. fetida*) (Carter et al. 2016a; González-Alcaraz et al. 2020).

In a study by Sidhu et al. (2019), earthworms were exposed to enrofloxacin, and BAFs were estimated for depurated (3.7) and non-depurated earthworms (18). These findings indicate that during the depuration of contaminated soil and the elimination phase of earthworm metabolism, approximately 81% of enrofloxacin is excreted. Additionally, in a study by Bao et al. (2018), it was observed that the accumulation of oxytetracycline was higher in horsebean plants than in earthworms, even when both were exposed to the same pollution. It is important to note that BAFs estimated under laboratory conditions represent a worst-case scenario, in which earthworms spend 100% of their time in contaminated soil. However, under field conditions, differences may arise due to heterogeneity and variations in the chemical stability and availability of pharmaceuticals (Bao et al. 2018). Furthermore, the incorporation of animal manure can enhance BAFs in earthworms (Song et al. 2022). Overall, BAFs can also be influenced by various factors such as the chosen estimation method, experimental design, organism studied, environmental context (whether aquatic or terrestrial), and physicochemical properties of the environment (Bao et al. 2018; Song et al. 2022).

### Earthworm exposure to pharmaceuticals in biochar-amended and non-amended soils

**Fig. 1:**
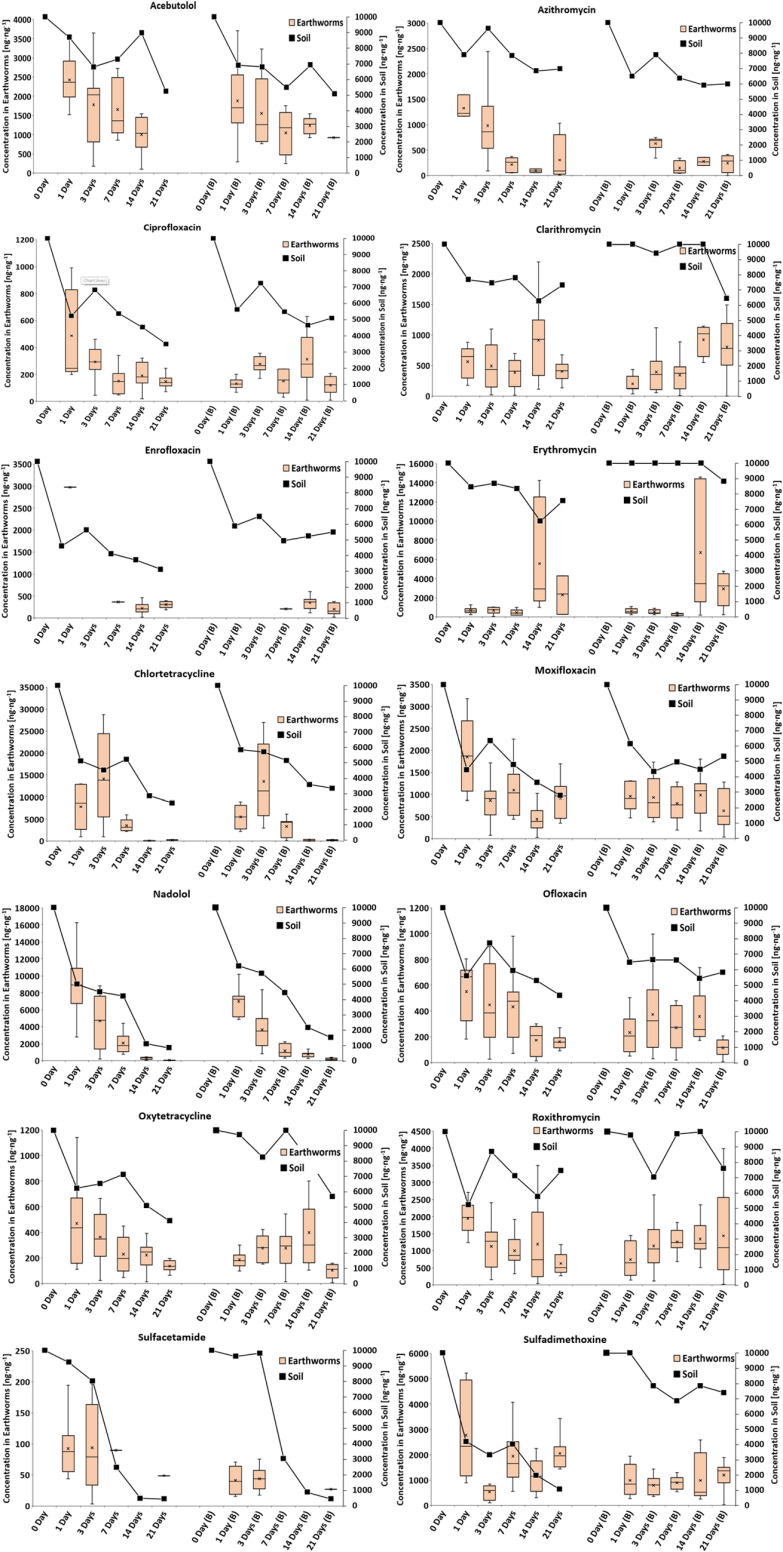

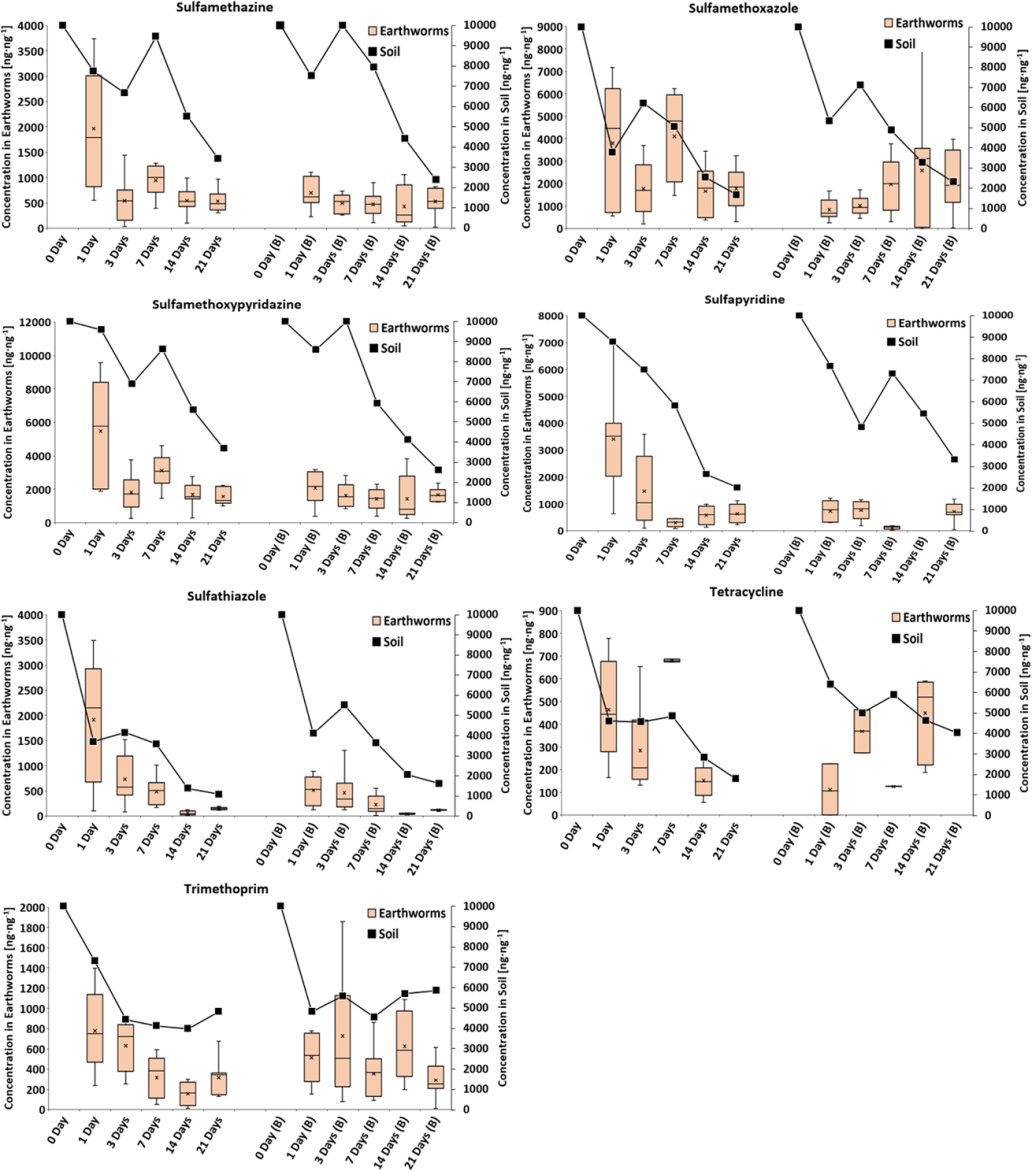
Concentrations of individual pharmaceuticals within the earthworms (left y axis; [ng g^−1^ dw]) and in soil (right y axis; [ng g^−^.^1^ dw]) over the 21 days, both in non-amended soil and biochar-amended soil (B)

The trends depicted in Fig. 1 (along with the heatmap in Fig. S1 for earthworm samples) illustrate a consistent decrease in both PhACs within earthworms and the surrounding soil over time, regardless of whether the soil was biochar-amended or non-amended, except clarithromycin and erythromycin (both belonging to Macrolides). As discussed earlier, PhAC concentrations within earthworm tissue are subject to various factors, leading to changes over the exposure period, primarily mirroring soil pollution levels. However, comparing these results with existing literature is challenging due to the limited number of studies available. The majority of studies typically conduct one-time sampling at the conclusion of the exposure experiment (Li et al. 2016; Carter et al. 2016b; Sidhu et al. 2019; Rivier et al. 2019; Ashworth et al. 2023; Grasserová et al. 2024) or solely collect earthworm samples during monitoring studies (Bergé and Vulliet 2015). In contrast to our findings, a study by Carter et al. 2014a observed increasing concentrations of carbamazepine and fluoxetine in earthworms during a 168 h exposure phase, followed by reaching a steady state. Conversely, in the same study, such behavior was not observed for diclofenac and orlistat, where the concentrations continuously increased without reaching a steady state by the end of the exposure phase. Additionally, a study from Wen et al. (2011) noted increasing concentrations of ciprofloxacin within earthworm tissue and described uptake kinetics using a first-order kinetic model. Furthermore, a study by Chen et al. (2020) reported decreasing concentrations of triclosan in soil, whereas concentrations within earthworm bodies increased during the exposure phase until reaching a steady state. Meanwhile, a study by Bergé and Vulliet (2015) conducted analyses of both real samples and laboratory experiments, though limit of detection (LoD) values were reported for the majority of laboratory samples, likely due to low soil contamination and metabolization of substances by earthworms. To the best of our knowledge, no similar study has reported decreasing concentrations of PhACs in earthworms alongside declining concentrations of these drugs in soil. This disparity with our results may be attributed to the use of different pharmaceuticals (and their physicochemical properties), sampling times, and overall experimental design differences, which vary considerably across studies. Experimental durations range from a few days to months (Li et al. 2016; Carter et al. 2016b; Sidhu et al. 2019; Rivier et al. 2019; Ashworth et al. 2023; Grasserová et al. 2024), with some studies encompassing both exposure and elimination phases (Wen et al. 2011; Carter et al. 2014a), while others expose earthworms to contaminated soil (Wen et al. 2011; Carter et al. 2014a; Chen et al. 2020), and some solely feed earthworms with contaminated vegetables (sulfamethoxazole, trimethoprim, sulfapyridine) (Ashworth et al. 2023).

The influence of biochar presence on the bioavailability of pharmaceuticals to earthworms was investigated using ANOVA with subsequent post hoc tests. Significant differences (*p* < 0.05) were revealed for 14 out of 21 pharmaceuticals, specifically azithromycin, ciprofloxacin, moxifloxacin, nadolol, ofloxacin, oxytetracycline, roxithromycin, sulfadimethoxine, sulfamethazine, sulfamethoxazole, sulfamethoxypyridazine, sulfapyridine, sulfathiazole, and tetracycline, after a 1-day exposure period. Although the uptake was notably reduced during the initial day due to the presence of biochar for some PhACs, no significant differences were noted in the subsequent days. Notably, studies (Williams et al. 2015; Li et al. 2020) have reported that incorporating 0.5–1.0% w/w of biochar in soil effectively reduces the bioavailability of PhACs to plants; however, this did not hold true for earthworms in our experiments. Additionally, a study by Li et al. (2020) has reported both increases and decreases in PhAC uptake by radish in the presence of 1.0% biochar. To our knowledge, no studies have been conducted on the soil-biochar-earthworms-pharmaceuticals system. Nevertheless, a study from Chen et al. 2023 investigating the influence of 0.0–5.0% biochar on the bioavailability of difenoconazole (a fungicide) to earthworms after 6 days yielded positive results, with uptake decreasing as the concentration of biochar in the soil increased. However, a review by Lin et al. (2022) reported conflicting results regarding the impact of biochar on the bioavailability of organic micropollutants to earthworms, attributable to various factors, including biochar properties, micropollutants, soil properties, and the type of soil organism. Several factors related to biochar can influence its sorption capacities and the fixation of pollutants, including the type of raw materials, specific surface area, preparation methods, pyrolysis temperature, pore structure, functional groups, nutrient composition, particle size, and degree of aging of biochar-based materials. The direct adsorption mechanisms of pristine biochar on organic pollutants involve pore filling, electrostatic interaction, hydrogen bonding, hydrophobic effects, and π-π interactions, which can be further enhanced through modifications (Lin et al. 2022). However, the limited efficacy of biochar observed in our study in lowering bioavailability only during the first day could be attributed to earthworms releasing intestinal secretions. These secretions serve to enhance the availability of nutrients, but in this case, they may inadvertently increase the bioavailability of pharmaceuticals as well. Unlike plants and microorganisms, earthworms can ingest soil particles and absorb soil contaminants through their digestive tracts, significantly affecting the bioaccumulation of organic contaminants. Moreover, as biochar ages, pollutants adsorbed onto biochar-based materials may be desorbed again over time (Lin et al. 2022). Other negative aspects of biochar in the soil environment include the release of polycyclic aromatic hydrocarbons (PAHs) into the soil (Wang et al. 2019) and prolonging the degradation of PhACs in terrestrial environments (Lin et al. 2022).

The influence of biochar on the degradation rate of PhACs in soil was statistically significant (*p* < 0.05) in the case of 16 out of 21 PhACs, as shown in Table 1. Specifically, the addition of 2.0% biochar to soil increased the degradation rate of azithromycin (from *k* = 0.021 d^−1^ to *k* = 0.032 d^−1^), sulfamethazine (from *k* = 0.045 d^−1^ to k = 0.063 d^−1^), and sulfamethoxypyridazine (from *k* = 0.045 d^−1^ to *k* = 0.0645 d^−1^) while decreasing the degradation rates of ciprofloxacin (from *k* = 0.056 d^−1^ to *k* = 0.043 d^−1^), clarithromycin (from *k* = 0.0336 d^−1^ to *k* = 0.020831 d^−1^), enrofloxacin (from *k* = 0.06 d^−1^ to *k* = 0.035 d^−1^), erythromycin (from *k* = 0.033 d^−1^ to *k* = 0.00592 d^−1^), nadolol (from *k* = 0.131 d^−1^ to *k* = 0.098 d^−1^), ofloxacin (from *k* = 0.044 d^−1^ to *k* = 0.033 d^−1^), oxytetracycline (from *k* = 0.0446 d^−1^ to *k* = 0.024 d^−1^), roxithromycin (from *k* = 0.0413 d^−1^ to *k* = 0.0120 d^−1^), sulfadimethoxine (from *k* = 0.1140 d^−1^ to *k* = 0.0153 d^−1^), sulfamethoxazole (from *k* = 0.091 d^−1^ to *k* = 0.075 d^−1^), sulfapyridine (from *k* = 0.082 d^−1^ to *k* = 0.050 d^−1^), tetracycline (from *k* = 0.088 d^−1^ to *k* = 0.049 d^−1^), and trimethoprim (from *k* = 0.045 d^−1^ to *k* = 0.030 d^−1^). Similar results were reported in a study (Li et al. 2020), in which the half-life of several pharmaceuticals in soil were prolonged with the addition of 1.0% biochar to the soil, whereas others were unaffected, depending on biochar and PhAC properties. The prolonged degradation time can be attributed to the incorporation of biochar, which amplifies the soil’s adsorption capacity, thereby decreasing the fraction of PhACs available in the pore space for bacteria and consequently reducing the biodegradation rate (Hurtado et al. 2017; García-Delgado et al. 2023). Meanwhile, some studies (Sun et al. 2022; Patel et al. 2022) have also reported an enhanced degradation rate because biochar encourages microbial colonization and activity by providing both carbon and other essential nutrients.

It is indispensable to note that soil characteristics, both physicochemical and biological, play an important role in the dissipation and degradation of organic micropollutants (Majumder et al. 2023). In agreement with previous studies (Song and Guo 2014; Pikkemaat et al. 2016; Pan and Chu 2016; Berendsen et al. 2021), the degradation values for pharmaceuticals fall within the ranges previously reported (Table 1). Under general conditions, most veterinary antibiotics are degradable in soil, with a half-life (DT_50_) of less than 30 days (e.g., sulfonamides), whereas certain antibiotics such as macrolides, tetracyclines, and fluoroquinolones can persist for over 120 days (Song and Guo 2014; Pikkemaat et al. 2016; Pan and Chu 2016; Berendsen et al. 2021).

### Ecotoxicological endpoints

**Fig. 2:**
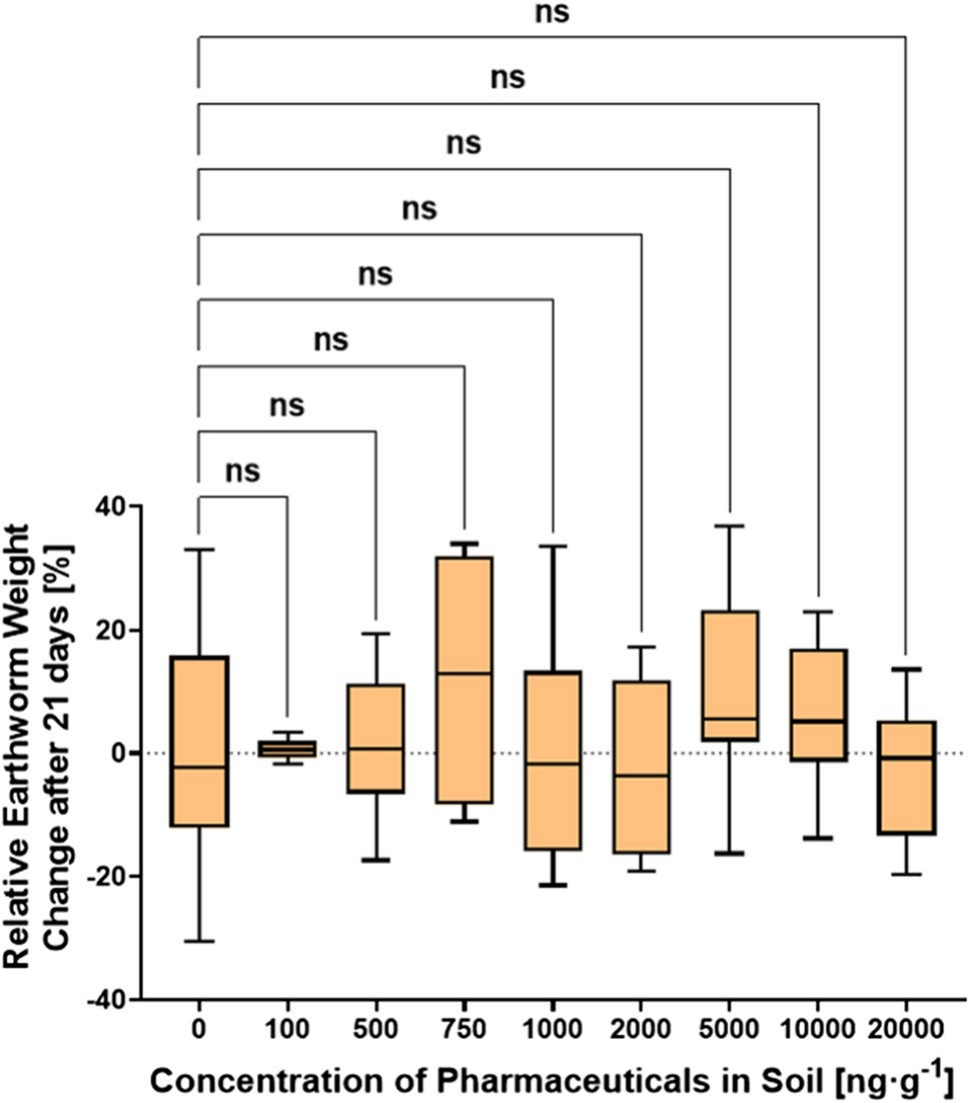
Relative weight change of earthworms after 21 days of exposure to different concentrations of pharmaceutical mixture in the terrestrial environment. Significant effects are indicated by asterisks (*) or “ns” for non-significant results

During the exposure of *E. fetida* to various concentrations of the PhAC mixture, no mortality was observed. Figure 2 illustrates the relative weight change of earthworms exposed to different concentrations of the pharmaceutical mixture in a terrestrial environment after 21 days. The relative weight change for depurated earthworms after 21 days ranged in boxplots Q1-Q3 from − 12.6 to 15.8% for the control experiment, consistent with a study from Grasserová et al. (2024). To determine whether the concentration of the pharmaceutical mixture in the soil had a significant effect on the relative weight loss or gain of earthworms, Dunnett’s test was performed. However, no significant effect was indicated (*p* > 0.05), which aligns with findings from previous studies (Bao et al. 2018; Yang et al. 2020). A study by Bao et al. (2018) also reported an insignificant effect of 200 μg∙g^−1^ oxytetracycline in soil on earthworm weight change. Similarly, a study by Yang et al. (2020) found no effect on *E. fetida* weight or mortality rate at ciprofloxacin soil concentrations of 10, 100, and 500 μg∙g^−1^ after 28 days. Further study by Pino et al. (2015) investigated the ecotoxicological effects of individual compounds from β-blockers, sulfonamides, and tetracyclines and found no lethal effect on *E. fetida* at soil concentrations below 2000 μg∙g^−1^, with a propranolol LC_50_ value of 3299 μg∙g^−1^. Similarly, a study by Li et al. (2015) exposed *E. fetida* to enrofloxacin at various concentrations ranging from 0 to 2.5 mg∙g^−1^, observing significant negative effects on growth at concentrations of 1 mg∙g^−1^, on reproduction at 0.5 mg∙g^−1^, and in avoidance test at 0.25 mg∙g^−1^. Additionally, a study from Lin et al. (2012) reported a significant negative effect of chlortetracycline on *E. fetida* reproduction at its soil concentration of 100 μg∙g^−1^. Overall, the results of ecotoxicological studies exposing earthworms to PhACs (from the perspective of weight changes and mortality rates) suggest that earthworms are not significantly affected by PhAC pollution at environmentally relevant concentrations, unlike other organisms in the soil, such as plants and soil microbiota. Although, studies (Dong et al. 2012; Lin et al. 2012; Wang et al. 2018; Yang et al. 2020) utilized DNA damage and changes in enzyme activities as biomarkers to evaluate the genotoxicity and oxidative stress of tetracyclines and fluoroquinolones on *E. fetida*, which are considered more sensitive biomarkers than relative weight changes. The findings (Dong et al. 2012) revealed that both antibiotics elicited substantial genotoxicity in *E. fetida*, exhibiting a dose-dependent and pharmaceutical-dependent pattern. Additionally, enzyme analysis highlighted further evidence of biochemical stresses induced by the antibiotics.

It is essential to acknowledge that during the experiment, the concentrations of PhACs declined due to their degradation, implying that earthworms were not consistently exposed to the same concentrations throughout the duration of exposure. In addition, each PhAC degrades at a different rate in the soil environment, as indicated in Table 1. Despite the decreasing concentrations of parent drugs, various PhAC metabolites are formed, which often remain unidentified and unquantified in available studies (Tanoue et al. 2012; Riemenschneider et al. 2017). Furthermore, most ecotoxicological studies have exposed earthworms to a single PhAC per experiment (Lin et al. 2012; Li et al. 2015; Bao et al. 2018), whereas our study conducted exposure experiments with a mixture of 21 drugs. Exposure of earthworms to a mixture of pharmaceuticals can result in negative synergistic effects of PhACs, thereby enhancing their toxicity. However, such exposure more closely resembles real environmental conditions, where mixtures of various organic micropollutants and PhACs (potentially including their metabolites) are found in terrestrial environments. Additionally, conducting exposure experiments with individual substances is nearly impossible because of their high quantity and increasing number.

**Fig. 3:**
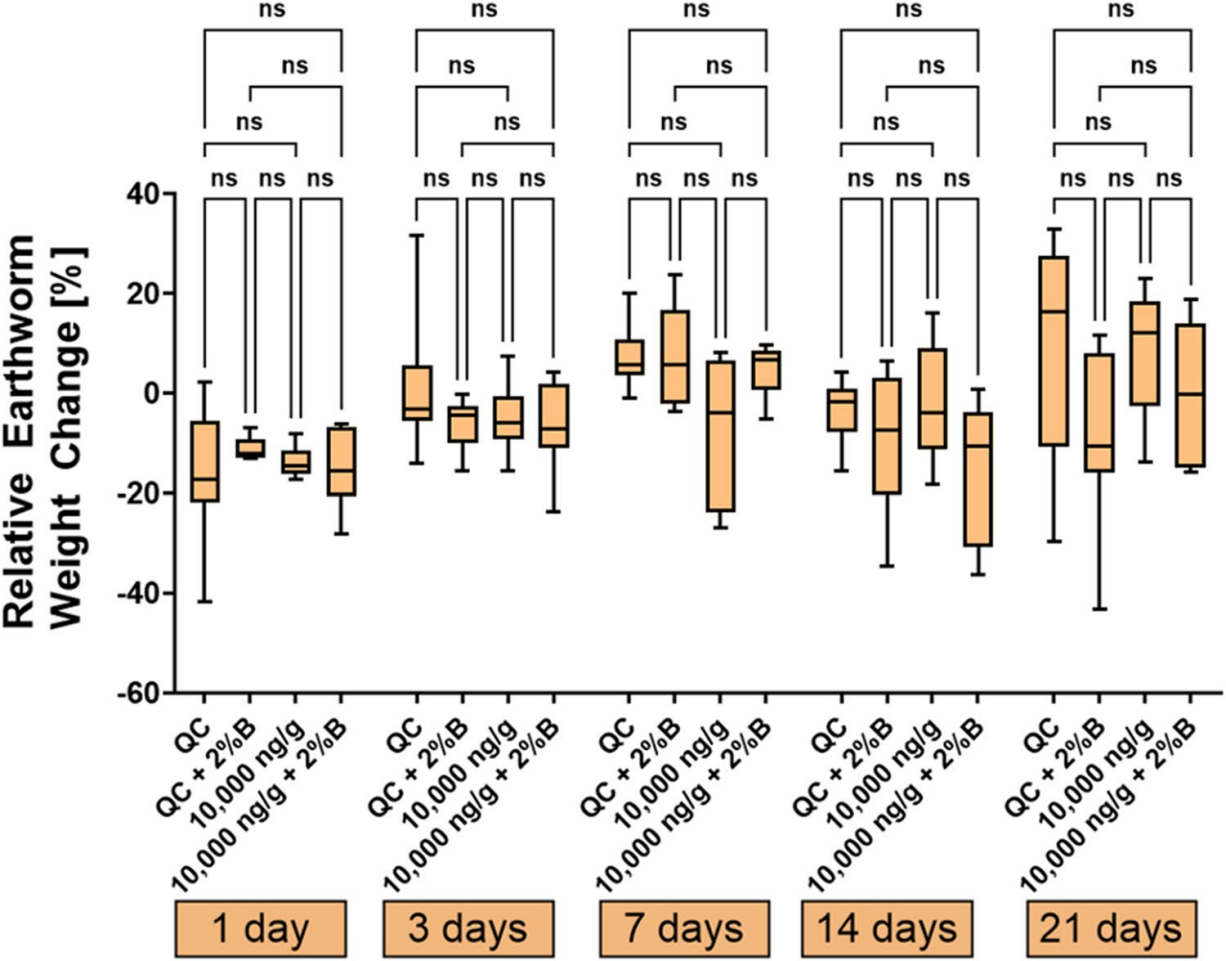
Relative weight change of earthworms during exposure to pharmaceutical mixture at a concentration of 10000 ng∙g^−1^ in non-amended and biochar-amended soil. Significant effects are indicated by asterisks (*) or “ns” for non-significant results

Furthermore, earthworms were exposed to a mixture of pharmaceuticals at a concentration of 10000 ng∙g^−1^ over the course of 21 days, either in non-amended soil or soil amended with biochar at a concentration of 2% (Fig. 3). As in the previous experiment, no earthworm mortality was observed. Additionally, there was no strong and statistically significant correlation observed between the relative weight change of earthworms and exposure time in across all treatment groups (*p* > 0.05). This lack of correlation may be attributed to the earthworm weight loss on day 14. ANOVA and Dunnett’s test did not reveal any statistically significant differences (*p* > 0.05) between the treatment groups over time. This suggests, similar to previous results with a range of different concentrations of the PhAC mixture, that earthworm relative weight change was not significantly influenced by PhAC pollution, the presence of 2% biochar, or their combination, even though biochar significantly affected the persistence of PhACs in soil. A study (Bean et al. 2016) reported earthworm-relative weight loss due to exposure to fluoxetine at a soil concentration of 600 μg∙g^−1^ after 21 days, whereas earthworms in the control group gained weight. Furthermore, to the best of our knowledge, no study has been carried out in a system involving soil-biochar-earthworms-pharmaceuticals to compare our results with.

#### Spatialdistribution ofveterinary antibiotics within earthworm tissue

Earthworms were subjected to a mixture of enrofloxacin (ENR, belonging to FQs), tetracycline (TC, belonging to TCs), and erythromycin (ERY, belonging to MLs), representing three distinct classes of veterinary antibiotics. These compounds were selected to encompass a wide range of molecular weights (ENR: 359.4, TC: 444.4, and ERY: 733.9). Additionally, these antibiotics exhibit varying bioaccumulation factors as shown in Table 1. Erythromycin has a high BAF of 0.317 ± 0.010, whereas enrofloxacin and tetracycline have lower BAFs of 0.0402 ± 0.0018 and 0.0467 ± 0.0014, respectively. The aim was to evaluate potential disparities in the uptake mechanism, uptake rate, and spatial distribution. Consequently, earthworm samples were collected at intervals of 1, 3, and 5 days following exposure to these veterinary antibiotics.

**Fig. 4:**
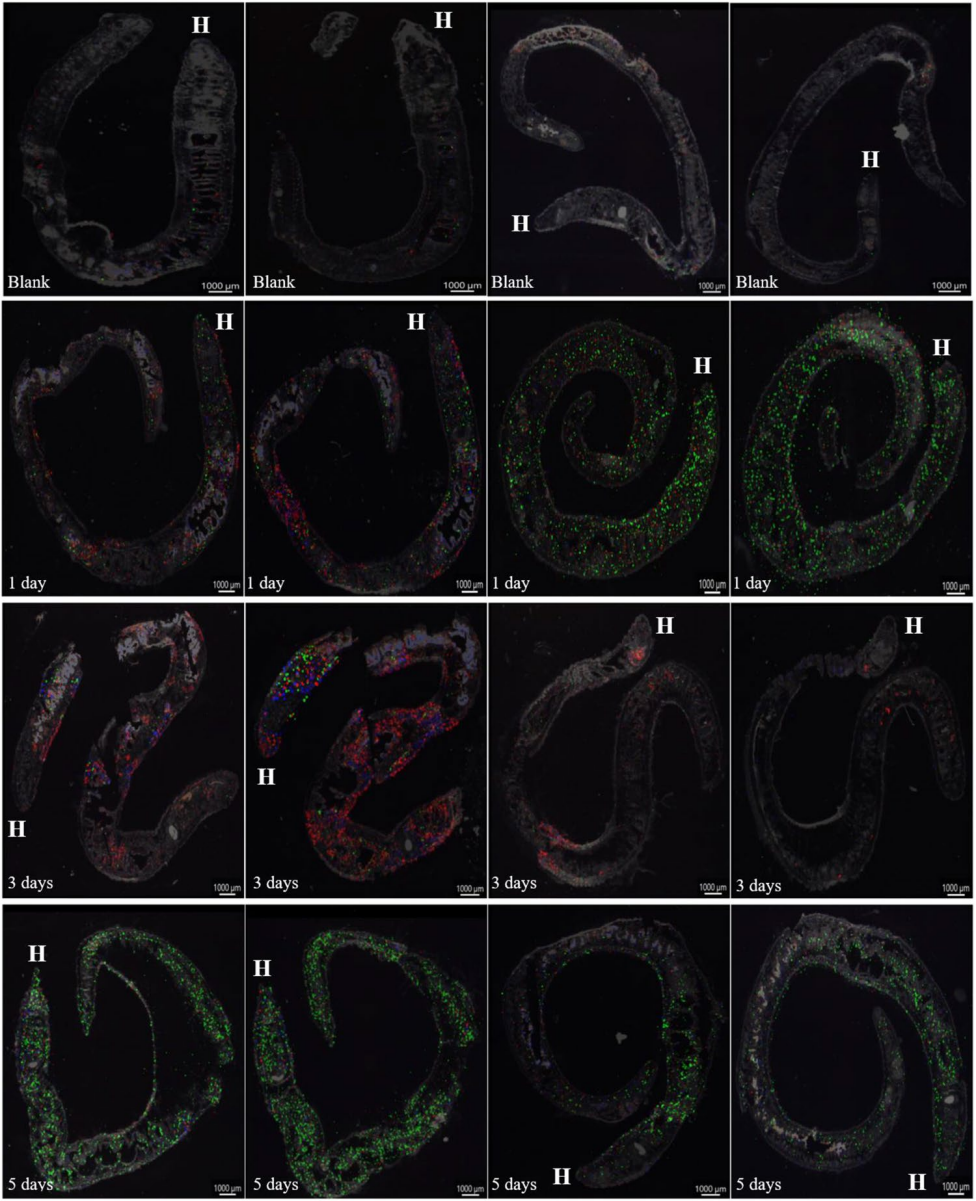
Spatial distribution of veterinary antibiotics in earthworm tissue visualized by blend MS images (ENR = Red; TC = Blue; and ERY = Green). Time of earthworm exposure to contaminated soil is noted in bottom left, and “H” Indicates earthworm head position

The mass spectra acquired via MALDI-MS were processed utilizing IMAGEREVEAL MS software. Subsequently, the data were normalized to the total ion count (TIC), with a threshold value set at 5 a.u. Targeted analysis for veterinary antibiotics was conducted with an m/z tolerance of 5 ppm. Notably, during MALDI-MS analysis, ENR occurred at m/z 360.1718 as [M + H]^+^, TC at m/z 445.1605 as [M + H]^+^, and ERY at m/z 734.4685 as [M + H]^+^. The outcomes of MALDI MSI yielded blend images (Fig. 4) combining ENR, TC, and ERY. However, individual images for each substance are presented in Fig. S2 to Fig. S5, along with their intensities. Transparency overlaid with optical microscope images was set to 0.75, for both blend (Fig. 4) and separate images (Fig. S2-S5).

The application of MALDI-MS to small molecules has often been deemed challenging or even impossible because of the intense and fluctuating matrix background. Despite these challenges, researchers have devised various technical solutions, including novel matrices and matrix deposition techniques, to generate mono-disperse layers of matrix/analyte crystals. However, MALDI-MS is still perceived as a non-quantitative technique by many researchers (Rzagalinski and Volmer 2017). Nevertheless, numerous quantitative MALDI-MSI strategies have been implemented, such as in-solution, on-tissue, or in-tissue quantitative approaches. Despite their utility, these approaches have limitations stemming from matrix effects, extraction efficiency, and required sample preparation (Lagarrigue et al. 2016; Rzagalinski and Volmer 2017; Boskamp and Soltwisch 2020). Consequently, LC–MS methodologies are often preferred in environmental studies for the quantification of low-molecular-weight micropollutants due to their ability to detect trace concentrations. However, MALDI-MS results are still valuable for gaining insights into the spatial distribution of targeted compounds (Villette et al. 2019; Davis et al. 2020), as also demonstrated in this study.

The MSI images (Fig. 4) align with the quantification data obtained via LC-TQ during the exposure of earthworms to the pharmaceutical mixture (Fig. 1). Both LC-TQ and MALDI-MS results indicate rapid uptake of drugs on the first day, which persists in subsequent days. Additionally, findings from both methods highlight ERY as the most abundant substance. Moreover, both the box plots (Fig. 1) and MSI images (Fig. 4) indicate considerable variation in PhAC uptake across earthworm samples, likely due to biological variability in *E. fetida*.

According to available studies (Roubalová et al. 2015; Diez‐Ortiz et al. 2015), soil contaminants can enter the earthworm body through two routes: (1) dermal exposure via contact with and transfer across the skin, and (2) ingestion followed by transfer across the gut epithelium. Research suggests that hydrophilic contaminants primarily enter the earthworm body through the skin, which is covered by secreted mucus, whereas hydrophobic substances enter via the digestive tract (Roubalová et al. 2015). Previous study (Diez‐Ortiz et al. 2015) on conventional chemicals has generally indicated that dermal exposure is the prevailing route. However, it is evident that earthworms possess a complex digestive system in which the earthworm and gut microbes mutually benefit from the degradation of ingested organic material and contribute to the degradation of contaminants (Rodriguez-Campos et al. 2014).

The MSI images (Fig. 4) indicate that antibiotic uptake likely involves a combination of pathways, as antibiotics are observed distributed throughout the entire body tissue of earthworms, rather than being confined to surface-proximal tissues or the digestive tract. Furthermore, variations in antibiotic abundances between different days are evident and could be attributed to varying uptake rates, earthworm metabolism, and biological variability within *E. fetida*. Moreover, no local concentration maxima were observed within the earthworm’s body, although this observation could be attributed to the relative simplicity of the *E. fetida* organism compared with other organisms, as indicated by results from other studies (Villette et al. 2019; Davis et al. 2020). Previous research has shown specific distribution patterns of pharmaceuticals in *Fathead minnows* based on their function (Davis et al. 2020), and diverse distributions for each pharmaceutical were observed in *Salix alba* leaves (Villette et al. 2019). Moreover, to the best of our knowledge, no study has yet reported the spatial distribution of veterinary antibiotics (VAs) within earthworm tissue.

Additionally, MSiReader software was employed to generate localized TIC images presenting distinct mass ranges and the number of analytes for each pixel of the acquired data (Fig. S6). Notably, within these images, the digestive tract emerges as the most prominent region, particularly notable in the mass-to-charge ratio (m/z) range of 600–1000, accompanied by the highest concentration of analytes. The observed variation in MSI intensity at the beginning of the digestive tract compared with other regions of the earthworm’s body can be attributed to a multitude of factors, including specialized functions, unique cell types, and microbial communities.

## Conclusion

This study provides valuable insights into the behavior of 21 PhACs within the soil-earthworm system. Bioaccumulation factors (BAFs) for earthworms were determined using a novel time-weighted average approach, considering both initial soil contamination and pharmaceutical degradation. After 21 days of exposure, BAF values ranged from 0.0253 to 0.329, indicating low uptake by *E. fetida*. These BAFs, calculated over a wide range of concentrations, enable the estimation of pharmaceutical concentrations within earthworm bodies based on soil contamination to asses associated environmental risks, emphasizing the critical role of earthworms in maintaining soil quality and supporting global food production.

Biochar had no significant effect on the bioavailability of pharmaceuticals to earthworms except on the first day (*p* < 0.05). However, biochar significantly prolonged the persistence of some pharmaceuticals (*p* < 0.05). No significant ecotoxicological effects on earthworm weight or mortality were observed (*p* > 0.05). On the contrary, other studies have shown that biochar can decrease the bioavailability of pharmaceutical residues in the environment. These conflicting findings underscore the need for further investigation to determine optimal biochar properties for managing pharmaceutical residues in soil environment.

Additionally, MSI analysis has indicated that pharmaceutical uptake by *E. fetida* can occur both through skin contact and ingestion, as pharmaceuticals were distributed throughout the entire earthworm tissue without specific localization. This analysis also serves as a proof of concept, as no method has yet been published for spatially analyzing small drug molecules within *E. fetida* after uptake from the terrestrial environment.

### Supplementary Information

Below is the link to the electronic supplementary material.

Supplementary file1 Supplementary information is available free of charge at Detailed information about Chemicals and pharmaceutical standards; physicochemical properties of selected pharmaceuticals, soil and biochar; Description of extractions methods for earthworm and soil samples; Detailed description of LC-MS/MS method including MRM transitions of selected pharmaceuticals; Heatmap of pharmaceutical distribution in earthworm samples over a 21-day period; Microscope pictures of earthworms; Spatial distribution of individual veterinary antibiotics (ENR, TC, and ERY); and TIC images of different mass ranges and Number of analytes in earthworm tissue. (DOCX 13847 KB)

## Data Availability

The data that support the findings of this study are available from the corresponding author, Jan Fučík, upon reasonable request.
