# Supplementary material for: Assessing earthworm exposure to a multi-pharmaceutical mixture in soil: unveiling insights through LC–MS and MALDI-MS analyses, and impact of biochar on pharmaceutical bioavailability
Source: Environ Sci Pollut Res Int. 2024 Jul 19;31(35):48351–68. doi: 10.1007/s11356-024-34389-1 (PMC11297825; doi:10.1007/s11356-024-34389-1)
Supplement: Supplementary file 1 — Supplementary file1 Supplementary information is available free of charge at Detailed information about Chemicals and pharmaceutical standards; physicochemical properties of selected pharmaceuticals, soil and biochar; Description of extractions methods for earthworm and soil samples; Detailed description of LC-MS/MS method including MRM transitions of selected pharmaceuticals; Heatmap of pharmaceutical distribution in earthworm samples over a 21-day period; Microscope pictures of earthworms; Spatial distribution of individual veterinary antibiotics (ENR, TC, and ERY); and TIC images of different mass ranges and Number of analytes in earthworm tissue. (DOCX 13847 KB) [file 11356_2024_34389_MOESM1_ESM.docx]

**Supplementary Information for**

**Assessing Earthworm Exposure to a Multi-Pharmaceutical Mixture in Soil: Unveiling Insights through LC-MS and MALDI-MS Analyses, and Impact of Biochar on** **Pharmaceutical Bioavailability**

Jan Fučík^1,*^, Rea Jarošová^2^, Andreas Baumeister^3^, Sascha Rexroth^3^, Jitka Navrkalová^1^, Marian Sedlář^4^, Helena Zlámalová Gargošová ^1^, Ludmila Mravcová^1^

*^1^ Institute of Chemistry and Technology of Environmental Protection, Faculty of Chemistry, Brno University of Technology, Purkyňova 118, 612 00 Brno, Czech Republic*

*^2^* Veterinary *Research Institute Brno, Hudcova 296/70 , 621 00 Brno, Czech Republic*

*^3^* *Shimadzu Europa GmbH, Albert-Hahn-Straße 6, 472 69 Duisburg, Germany*

*^4^* *CEITEC Brno University of Technology, Purkyňova 656/123, 612 00 Brno, Czech Republic*

*corresponding author: [*xcfucikj@vutbr.cz*](mailto:xcfucikj@vutbr.cz)

**Table of Contents**

**Appendix 1** Chemicals and pharmaceutical standards

**Table S1** Physico-chemical properties of pharmaceuticals

**Table S2** Sampling location and physico-chemical properties of soil

**Table S3** Physico-chemical properties of biochar

**Appendix 2** Extraction Methods

**Appendix 3** Analytical method

**Table S4** MRM transitions of selected pharmaceuticals

**Fig. S1** Heatmap of pharmaceutical distribution in earthworm samples over a 21-day period

**Fig. S2** Microscope pictures of earthworms

**Fig. S3** Spatial distribution of Enrofloxacin within earthworm tissue

**Fig. S4** Spatial distribution of Tetracycline within earthworm tissue

**Fig. S5** Spatial distribution of Erythromycin within earthworm tissue

**Fig. S6** TIC of different mass ranges and Number of Analytes in earthworm tissue

**This document contains in total:** 15 pages; 4 Tables, 6 Figures

**Appendix 1 Chemicals and pharmaceutical standards**

**Chemicals and material for sample extraction and analysis**

Ethylenediamine tetraacetic acid (EDTA, ≥99%), citric acid monohydrate (≥99%), disodium hydrogen phosphate dodecahydrate (≥99%), and sodium sulfate anhydrous (≥95%), Potassium dihydrogen phosphate (≥99%) and hydrochloric acid (35%) were purchased from Lach-ner, s.r.o. (Czech Republic). Magnesium nitrate hexahydrate (>99%), ammonium (25%), methanol (LC-MS grade), acetonitrile (LC-MS grade), and water (LC-MS grade) were purchased from VWR (USA). Sodium hydroxide (>98%) and phosphoric acid (85%) were purchased from Penta Chemicals (Czech Republic). Formic acid (LC-MS grade), and sodium chloride (>99%) were purchased from Sigma Aldrich (Germany).

For QuEChERS, dispersive SPE (dSPE): DSC-18 SPE, and PSA SPE were purchased from Sigma Aldrich (Germany). Nitrogen gas (4.7) and argon gas (5.0) were purchased from SIAD Czech spol. s.r.o. (Czech Republic). Nylon syringe filters (13 mm, 0.22 μm) and solid phase extraction (SPE) HLB cartridges (200 mg/6 mL, particle diameter 25-35 μm) were purchased from Chromservis (Czech Republic).

**Chemicals and consumables for cryosectioning and MALDI-Imaging-HRMS**

ITO-glass Slides (0.7 mm, 25x75 mm) for MALDI Imaging were purchased from Bruker (Germany). Embedding medium Tissue Tekk OCT was purchased from Sakura Finetek (USA). Ethanol (LC grade) was purchased from VWR (USA). 2,5-Dihydroxybenzoic acid (98%), was purchased from Sigma Aldrich (Germany). Trifluoroacetic acid (LC-MS) was purchased from Biosolve (Netherlands).

**Pharmaceutical standards**

The following pharmaceuticals (see their properties in Table S1) were used: Acebutolol hydrochloride (≥99%), oxytetracycline hydrochloride (≥94%), and sulfacetamide (≥98%) were purchased from Honeywell (USA). Ofloxacin (≥98%), and sulfamethoxypyridazine (≥97%) were purchased from Thermo Fisher Scientific (USA). Azithromycin (≥98%), chlortetracycline hydrochloride (≥91%), ciprofloxacin (≥98%), clarithromycin (≥97%), enrofloxacin (≥99%), erythromycin (≥97%), moxifloxacin (≥96%), nadolol (≥98%), roxithromycin (≥95%), sulfadimethoxine (≥98%), sulfamethazine (≥99%), sulfamethoxazole (≥98%), sulfapyridine (≥99%), sulfathiazole (≥99%), tetracycline (≥98%) and trimethoprim (≥98%) were purchased from Sigma Aldrich (Germany).

The following substances were used as internal standards (IS): atenolol-d7 (≥97%), ciprofloxacin-d8 (≥99%), enrofloxacin-d5 (≥99%), spiramycin (≥90%) and trimethoprim-d9 (≥97%)were purchased from Sigma Aldrich (Germany). Sulfamethoxazole-d4 was purchased from Neochema GmbH (Germany). Sulfathiazole-d^4^ was purchased from Toronto Research Chemicals (Canada).

**Table S1** Physico-chemical properties of pharmaceuticals (values sourced from pubchem)

| **Pharmaceutical group** | **Substance name** | **Chemical formula** | **Mw [-]** | **pKa [-]** | **log P [-]** | **Solubility in water [mg∙L^-1^]** |
| --- | --- | --- | --- | --- | --- | --- |
| BETA BLOCKING AGENTS | Nadolol | C_17_H_27_NO_4_ | 309.4 | 9.7 | 0.7 | 46.4 |
|  | Acebutolol | C_18_H_28_N_2_O_4_ | 336.4 | 9.5 | 1.7 | 259 |
| TETRACYCLINES | Chlortetracycline | C_22_H_23_ClN_2_O_8_ | 478.9 | 7.4 | -0.62 | 0.259 |
|  | Oxytetracycline | C_22_H_24_N_2_O_9_ | 460.4 | 3.3; 9.5 | -1.6 | 47 |
|  | Tetracycline | C_22_H_24_N_2_O_8_ | 444.4 | 3.3; 7.7 | -2 | 231 |
| SULFONAMIDES AND TRIMETHOPRIM | Trimethoprim | C_14_H_18_N_4_O_3_ | 290.3 | 7.1 | 0.9 | 400 |
|  | Sulfamethazine | C_12_H_14_N_4_O_2_S | 278.3 | 2.7; 7.7 | 0.3 | 1,500 |
|  | Sulfapyridine | C_11_H_11_N_3_O_2_S | 249.3 | 8.4 | 0 | 33.1 |
|  | Sulfathiazole | C_9_H_9_N_3_O_2_S_2_ | 255.3 | 2.2; 7.2 | 0.1 | 373 |
|  | Sulfamethoxazole | C_10_H_11_N_3_O_3_S | 253.3 | 1.6; 5.7 | 0.9 | 610 |
|  | Sulfadimethoxine | C_12_H_14_N_4_O_4_S | 310.3 | 1.99; 6.91 | 1.6 | 343 |
|  | Sulfamethoxypyridazine | C_11_H_12_N_4_O_3_S | 280.3 | 2.02; 6.84 | 0.3 | 0.325 |
|  | Sulfacetamide | C_8_H_10_N_2_O_3_S | 214.2 | 2.14; 4.3 | -1.0 | 32.1 |
| MACROLIDES | Erythromycin | C_37_H_67_NO_13_ | 733.9 | 8.9 | 2.7 | 4.2 |
|  | Roxithromycin | C_41_H_76_N_2_O_15_ | 837.0 | 9.3 | 1.7 | 0.0189 |
|  | Clarithromycin | C_38_H_69_NO_13_ | 748.0 | 9.0 | 3.2 | 0.33 |
|  | Azithromycin | C_38_H_72_N_2_O_12_ | 749.0 | 8.5 | 4 | 2.37 |
| FLUOROQUINOLONE ANTIBACTERIALS | Ofloxacin | C_18_H_20_FN_3_O_4_ | 361.4 | 6.0; 9.3 | -0.4 | 28,300 |
|  | Ciprofloxacin | C_17_H_18_FN_3_O_3_ | 331.3 | 6.1; 8.7 | -1.1 | <1 |
|  | Enrofloxacin | C_19_H_22_FN_3_O_3_ | 359.4 | 5.55; 7.24 | -0.2 | 53.9 |
|  | Moxifloxacin | C_21_H_24_FN_3_O_4_ | 401.4 | 6.3; 9.1 | 0.6 | 1,146 |

**Table S2** Sampling location and physico-chemical properties of soil

| **Sampling location** | |
| --- | --- |
| State | Czech Republic |
| Region | The Vysočina region |
| Town | Jemnice |
| Sampling depth [cm] | 0-25 |
| **Physico-chemical properties of soil** | |
| Soil texture | Sandy Loam |
| Soil type | Fluvisol |
| Sand [%] | 52.05 |
| Silt [%] | 32.65 |
| Clay [%] | 15.30 |
| pH _(CaCl2)_ [-] | 6.47 |
| pH _(H2O)_ [-] | 7.37 |
| EC [mS∙cm^-1^] | 0.287 |
| Maximal water holding capacity [%] | 44 |
| Exchangable Mg^2+^ [mg∙kg-1] | 57.1 |
| Exchangable Ca^2+^ [mg∙kg^-1^] | 388.2 |
| Exchangable K^+^ [mg∙kg^-1^] | 135.8 |
| Exchangable Na^+^ [mg∙kg^-1^] | 45.0 |
| Exchangable NH_4_^+^ [mg∙kg^-1^] | 6.8 |
| Exchangable NO_3_^-^ [mg∙kg^-1^] | 189.0 |
| Exchangable PO_4_^3-^ [mg∙kg^-1^] | 194.2 |
| Total phosphorus – water extract [mg∙kg^-1^] | 46.7 |
| Total Nitrogen – water extract [%] | 0.128 |
| Total Carbon [%] | 2.50 |
| Inorganic Carbon [%] | 0.27 |
| Organic carbon [%] | 2.23 |
| Organic matter [%] | 3.83 |
| Ca - Aqua regia [mg∙kg^-1^] | 388.2 |
| K - Aqua regia [mg∙kg^-1^] | 1,594 |
| Mg - Aqua regia [mg∙kg^-1^] | 7,158 |
| Na - Aqua regia [mg∙kg^-1^] | 87.1 |
| NH_4_^+^ - Aqua regia [mg∙kg^-1^] | 89.6 |
| Total phosphorus - Aqua regia [mg∙kg^-1^] | 2,822 |
| PO_4_^3-^ - Aqua regia [mg∙kg^-1^] | 1,973 |

**Table S3** Physico-chemical properties of biochar (data available from study by Holatko et al. 2023)

| **Physico-chemical properties**  **of biochar** | |
| --- | --- |
| Dry matter content [%] | 65-75 |
| BET [m^2^∙g^-1^] | 289 |
| Ash_550°C_ [%] | 11.7 |
| Total Nitrogen [g∙kg^-1^] | 13.2 |
| Total Carbon [g∙kg^-1^] | 866 |
| Organic Carbon [g∙kg^-1^] | 74 |
| P [g∙kg^-1^] | 6.2 |
| K [g∙kg^-1^] | 24.4 |
| Ca [g∙kg^-1^] | 8.1 |
| Mg [g∙kg^-1^] | 6.7 |

**Appendix 2 Extraction Methods**

**Extraction of Pharmaceuticals from Earthworm Samples**

The PhACs from earthworm samples were extracted using our own already validated and published QuEChERS method (Mravcová et al. 2024). Briefly, 0.1 g of lyophilized and homogenized earthworms was weighed in a 50 ml PE centrifugation tube. Subsequently, 10 mg of EDTA was weighed and ceramic homogenizers were added, followed by pipetting of 5 ml of the extraction medium (MeOH:McIlvaine buffer pH 2.6 in a ratio of 80:20). Following this, the sample was vortexed for 1 min. Subsequently, separation salts (2 g anhydrous Na_2_SO_4_ and 0.5 g NaCl) were added, followed by vortexing for 1.5 min and centrifugation (3,500 rpm) for 10 min at 20 °C. In the purification step, after centrifugation, 2 ml of the organic phase was carefully pipetted into a 15 ml PE centrifugation tube preloaded with dSPE sorbents (12.5 mg DSC-18, 12.5 mg PSA, and 225 mg of anhydrous Na_2_SO_4_). The sample was vortexed for 1 min and centrifuged (3,500 rpm) for an additional 10 min at 20 °C. Finally, the sample was filtered through 0.22 μm nylon syringe filters (diameter 13 mm) into a 2-ml glass vial, ready for LC-MS/MS analysis.

**Extraction of Pharmaceuticals from Soil Samples**

The PhACs from soil samples were extracted using our own already validated and published method (Mravcová et al. 2024). Briefly, 1 g of soil was precisely weighed and placed into a 50 ml polyethylene (PE) centrifugation tube. **The extraction procedure (Steps 1-2)** involved pipetting 5 ml of methanol (MeOH) and 5 ml of phosphate buffer (pH 3) into the sample, followed by vortexing for 30 s. Subsequently, PhACs were extracted using an ultrasound bath for 10 min at 12°C. After sonication, the mixture was centrifuged at 4,800 rpm for 8 min at 20 °C. The resulting supernatant was transferred into a 30-ml dark glass vial. The extraction process was then repeated using the same extraction medium and extraction conditions. The extracts obtained from extraction rounds (1-2) were combined and placed in the same vial. In the subsequent **extraction steps (3-4)**, 0.6 g of EDTA was added to the soil along with 7.5 ml of acetonitrile (ACN), 7.5 ml of McIlvaine buffer (pH 8), 4.8 ml of Mg(NO_3_)_2_∙6H_2_O aqueous solution (concentration 0.5 g∙mL^-1^), and 0.2 ml of 2.5% NH_3_ aqueous solution in the centrifugation tube. The mixture was vortexed for 30 s, followed by PhAC extraction using an ultrasound bath for 10 min at 35°C. After sonication, the solution was centrifuged at 4,800 rpm for 8 min at 20 °C. Subsequently, the supernatant was transferred to a dark 30 mL glass vial. The extraction process was repeated with half the volume of the extraction medium (EM) without the addition of EDTA (3.75 ml of McIlvaine buffer, 3.75 ml of ACN, 2.4 ml of Mg(NO_3_)_2_ solution, and 0.1 ml of 2.5% NH_3_) under the same extraction conditions. The extracts obtained from extraction rounds (3-4) were combined and placed in the same vial. During the **pre-concentration step**, both vials were subjected to evaporation under a nitrogen stream in a thermostatic metal block heated to 40°C until each vial’s weight loss reached 6 g. Subsequently, the contents of both vials were combined and transferred into a 600-ml beaker, and the soil extract was diluted by adding 480-ml Milli-Q water to decrease the percentage of the organic phase (no additional pH adjustment was made). **In the solid phase extraction step**, the pre-concentrated soil extracts were purified using OASIS HLB cartridges (200mg; 6 ml; Particle diameter 25-35 μm; Chromservis, Czech Republic) using a Baker vacuum system (J.T. Baker, Deventer, The Netherlands). To outline the procedure briefly, the SPE column was conditioned with 6 ml of MeOH, followed by 6 ml of Milli-Q water, with a flow rate of approximately 1 ml∙min^-1^. Subsequently, the diluted soil extract was loaded onto the column at a flow rate of 5 ml∙min^-1^.The washing step was performed with 15 ml of Milli-Q water at a flow rate of 1 ml∙min^-1^, followed by 2 min of vacuum drying of the sorbents. Finally, the elution of PhACs was achieved by passing 9 ml of 0.1% formic acid (FA) in MeOH, and the eluate was collected into 20 ml glass vials. This was followed by evaporation under the nitrogen stream in the thermostatic metal block heated to 40°C to dryness. Subsequently, 5 μl of an internal standards mixture (concentration of mixture 10 μg∙mL^-1^) was introduced, followed by the addition of 995 μl of 0.1% FA in H_2_O:ACN (95:5). The sample was filtered through 0.22 μm nylon syringe filters (diameter 13 mm) into a 2-ml glass vial. The prepared sample was then subjected to LC-MS/MS analysis.

**Appendix 3 Annalytical Method**

**LC-MS/MS Method**

Earthworm and soil extracts were analyzed using our own already validated and published method for over 40 pharmaceuticals by (Mravcová et al. 2024). However, in this study, only 21 pharmaceuticals were analyzed, which allowed for lower limits of detection (LoDs) and quantification (LoQs) to be achieved, while maintaining similar recovery rates, as the extraction method remained the same.

Instrumental analysis for the quantification of PhACs in all extracts (soil and earthworms) was performed using ultra-performance liquid chromatography (UHPLC Agilent 1290 Infinity LC) coupled with a triple quadrupole mass spectrometer (Bruker EVOQ LC-TQ) with electrospray ionization (ESI). The gas sources of nitrogen and air were provided by an external gas generator (Peak Scientific – Genius 3045).

Chromatographic separation was accomplished using a Luna® Omega Polar C18 Phenomenex column (100 x 2.1 mm, 1.6 µm). The column temperature was optimized at 35°C, and the flow rate was set to 0.5 mL∙min^-1^. The mobile phases consisted of A) 0.1% FA in H_2_O and B) ACN, following a gradient program for the A eluent (%): t(0 min) = 90, t(0.5 min) = 90, t(13.0 min) = 35, t(14.0 min) = 10, and t(15.5 min) = 90. The LC method was set to a stop time of 16 min, with a 2-min re-equilibration time. The injection volume for all analyses was 7 µL. To prevent carry-over, an external needle wash was performed using a wash solvent composed of FA:H_2_O:ACN at a ratio of 1:9:90 for 30 s.

The MS conditions were set as follows for electrospray ionization in positive mode: spray voltage: 4,500 V; cone temperature: 350 °C; cone gas flow: 15 arbitrary units (a.u.); heated probe temperature: 500 °C; probe gas flow: 25 a.u.; nebulizer gas flow: 45 a.u.; and exhaust gas: ON. For both quantitative and qualitative analysis of PhACs, the multiple reaction monitoring (MRM) mode was employed, using the specific MRM transitions outlined in Table S4. Argon served as the collision gas at a pressure of 1.5 mTorr.

**Table S4** MRM transitions of selected pharmaceuticals

| **Analyte name** | **RT**  **[min]** | **Quantitative transition** | | | **Quantitative transition** | | | **Internal standards** |
| --- | --- | --- | --- | --- | --- | --- | --- | --- |
|  |  | **Precursor [m/z]** | **Productn[m/z]** | **CE [eV]** | **Precursor [m/z]** | **Productn[m/z]** | **CE [eV]** |  |
| Acebutolol | 3.65 | 337.2 | 116.0 | 20.0 | 337.2 | 319.0 | 10.0 | Atenolol-d7 |
| Azithromycin | 4.84 | 375.2 | 591.3 | 10.0 | 375.2 | 158.0 | 20.0 | Spiramycin |
| Chlortetracycline | 4.39 | 479.0 | 462.3 | 10.0 | 479.0 | 443.7 | 20.0 | none |
| Ciprofloxacin | 3.35 | 332.4 | 314.2 | 10.0 | 332.4 | 288.2 | 10.0 | Ciprofloxacin-d8 |
| Ciprofloxacin-d8 | 3.35 | 340.4 | 322.1 | 15.0 | 340.4 | 296.1 | 15.0 | - |
| Clarithromycin | 7.54 | 748.5 | 158.1 | 20.0 | 748.5 | 590.4 | 10.0 | Spiramycin |
| Enrofloxacin | 3.78 | 360.4 | 316.2 | 10.0 | 360.4 | 342.2 | 20.0 | Enrofloxacin-d5 |
| Enrofloxacin-d5 | 3.78 | 365.4 | 321.0 | 10.0 | 365.4 | 346.8 | 20.0 | - |
| Erythromycin | 6.57 | 734.5 | 158.2 | 30.0 | 734.5 | 576.3 | 10.0 | Spiramycin |
| Moxifloxacin | 4.83 | 402.0 | 358.2 | 20.0 | 402.0 | 384.2 | 20.0 | Enrofloxacin-d5 |
| Nadolol | 2.50 | 310.2 | 253.8 | 10.0 | 310.2 | 200.8 | 20.0 | Atenolol-d7 |
| Ofloxacin | 3.25 | 362.2 | 318.1 | 10.0 | 362.2 | 261.1 | 20.0 | Enrofloxacin-d5 |
| Oxytetracycline | 2.85 | 461.0 | 426.2 | 20.0 | 461.0 | 443.2 | 5.0 | none |
| Roxithromycin | 8.03 | 837.5 | 679.5 | 20.0 | 837.5 | 157.9 | 30.0 | Spiramycin |
| Spiramycin | 4.78 | 422.8 | 699.5 | 5.0 | 422.8 | 540.5 | 5.0 | - |
| Sulfacetamide | 1.62 | 215.2 | 156.0 | 5.0 | 215.2 | 108.2 | 10.0 | Sulfamethoxazole-d4 |
| Sulfadimethoxine | 5.48 | 255.0 | 156.0 | 10.0 | 255.0 | 92.1 | 15.0 | Sulfamethoxazole-d4 |
| Sulfamethazine | 3.02 | 279.3 | 186.0 | 10.0 | 279.3 | 92.0 | 30.0 | Sulfamethoxazole-d4 |
| Sulfamethoxazole | 4.14 | 254.3 | 156.0 | 10.0 | 254.3 | 108.1 | 20.0 | Sulfamethoxazole-d4 |
| Sulfamethoxazole-d4 | 4.12 | 258.3 | 159.8 | 10.0 | 258.3 | 96.2 | 20.0 | - |
| Sulfamethoxypyridazine | 3.20 | 281.1 | 156.0 | 10.0 | 281.1 | 92.2 | 20.0 | Sulfamethoxazole-d4 |
| Sulfapyridine | 1.97 | 250.3 | 156.0 | 10.0 | 250.3 | 92.2 | 20.0 | Sulfamethoxazole-d4 |
| Sulfathiazole | 2.27 | 256.0 | 156.0 | 10.0 | 256.0 | 92.2 | 20.0 | Sulfathiazole-d4 |
| Sulfathiazole-d4 | 2.23 | 260.0 | 96.0 | 20.0 | 260.0 | 160.0 | 10.0 | - |
| Tetracycline | 5.00 | 445.0 | 427.2 | 10.0 | 445.0 | 428.0 | 10.0 | none |
| Trimethoprim | 2.68 | 291.2 | 230.1 | 20.0 | 291.2 | 261.0 | 20.0 | Trimethoprim-d9 |
| Trimethoprim-d9 | 2.68 | 300.1 | 123.0 | 20.0 | 300.1 | 233.8 | 20.0 | - |


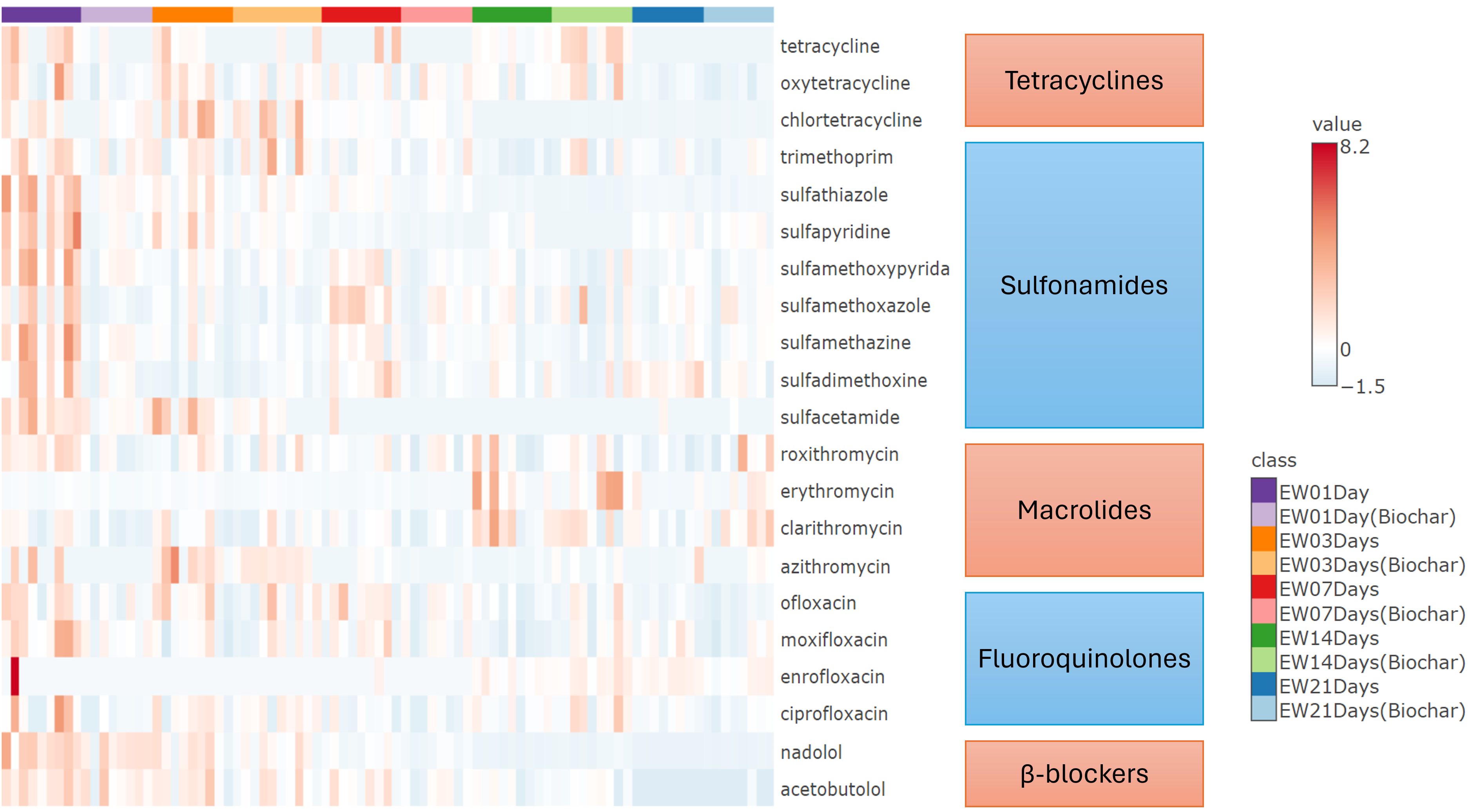


**Fig. S1** Heatmap of pharmaceutical distribution in earthworm samples over a 21-day period (created in MetaboAnalyst (Xia et al. 2009))

**
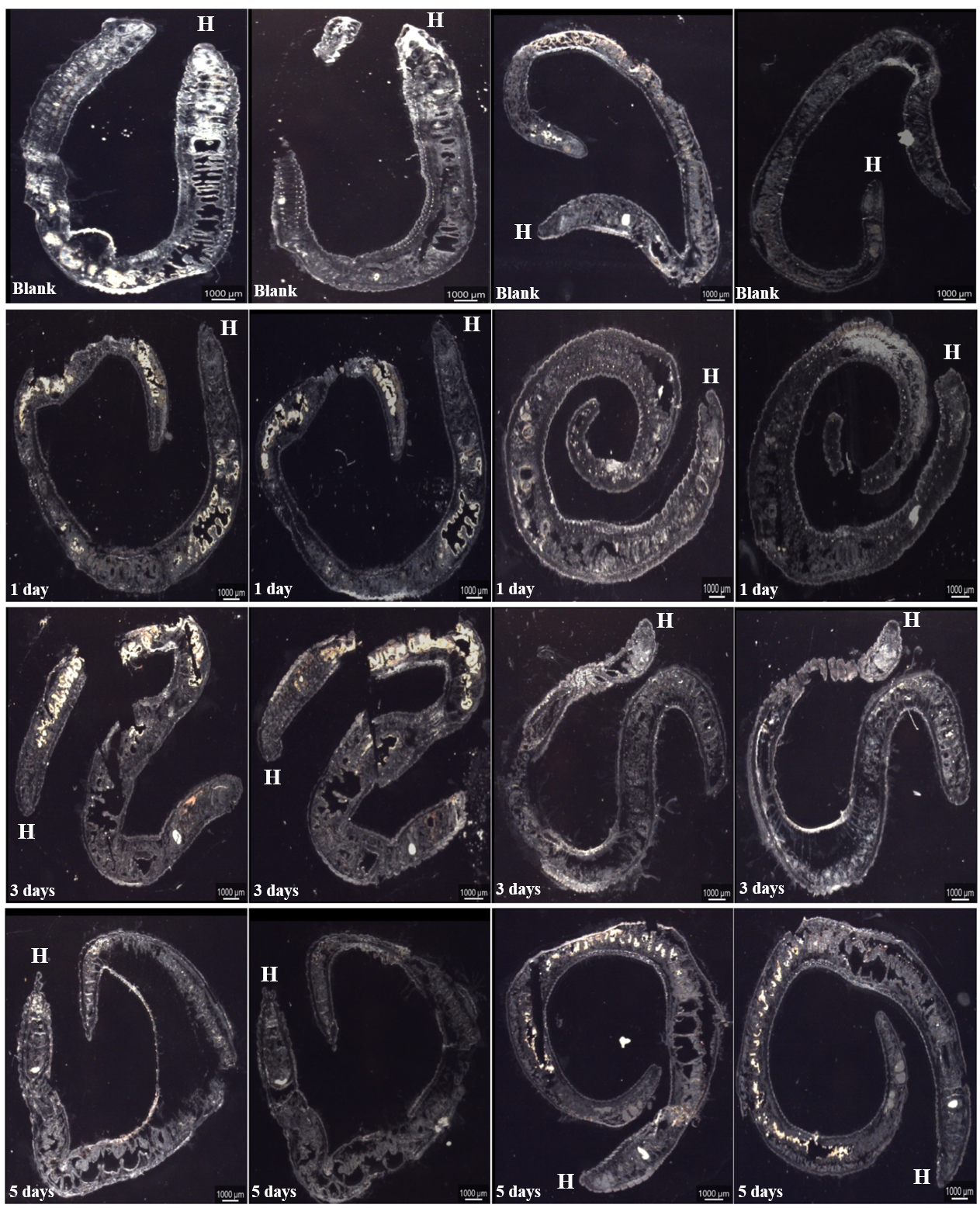
**

**Fig. S2** Microscope pictures of earthworm tissue. Time of earthworm exposure to contaminated soil is noted in Bottom Left, and 'H' Indicates Earthworm Head Position."

**
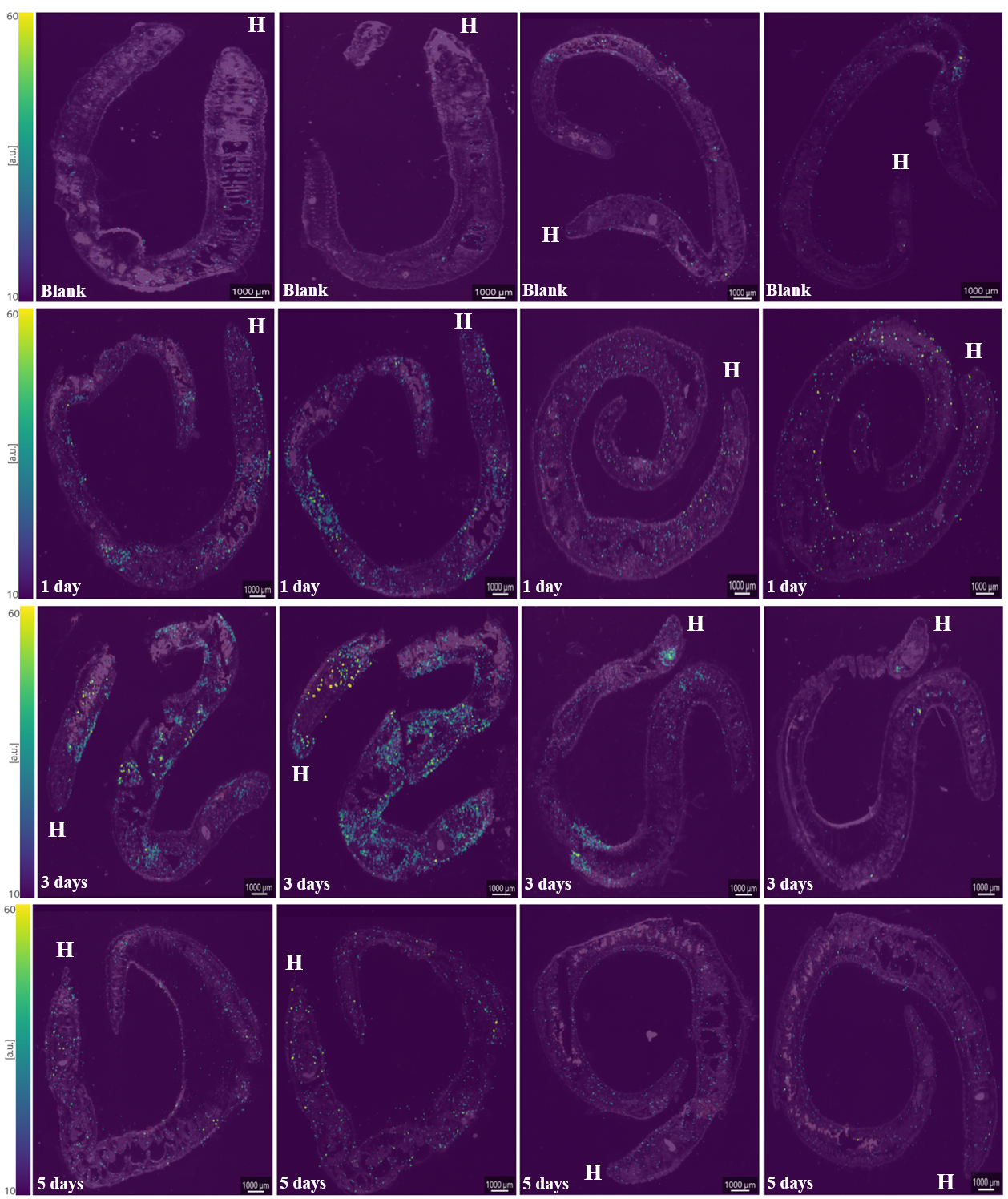
**

**Fig. S3** Spatial distribution of Enrofloxacin within earthworm tissue. Time of earthworm exposure to contaminated soil is noted in Bottom Left, and 'H' Indicates Earthworm Head Position."

**
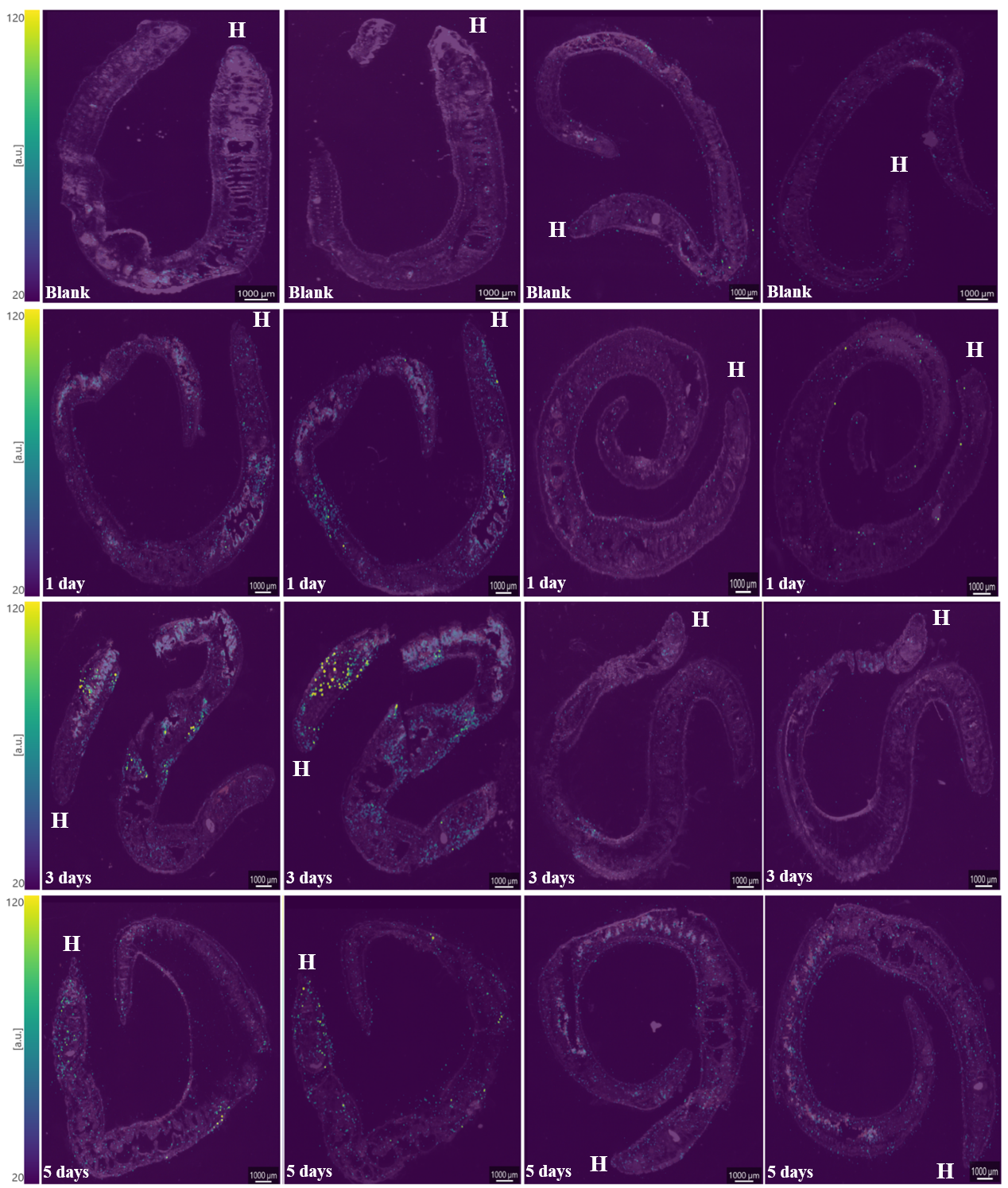
**

**Fig. S4** Spatial distribution of Tetracycline within earthworm tissue. Time of earthworm exposure to contaminated soil is noted in Bottom Left, and 'H' Indicates Earthworm Head Position."

**
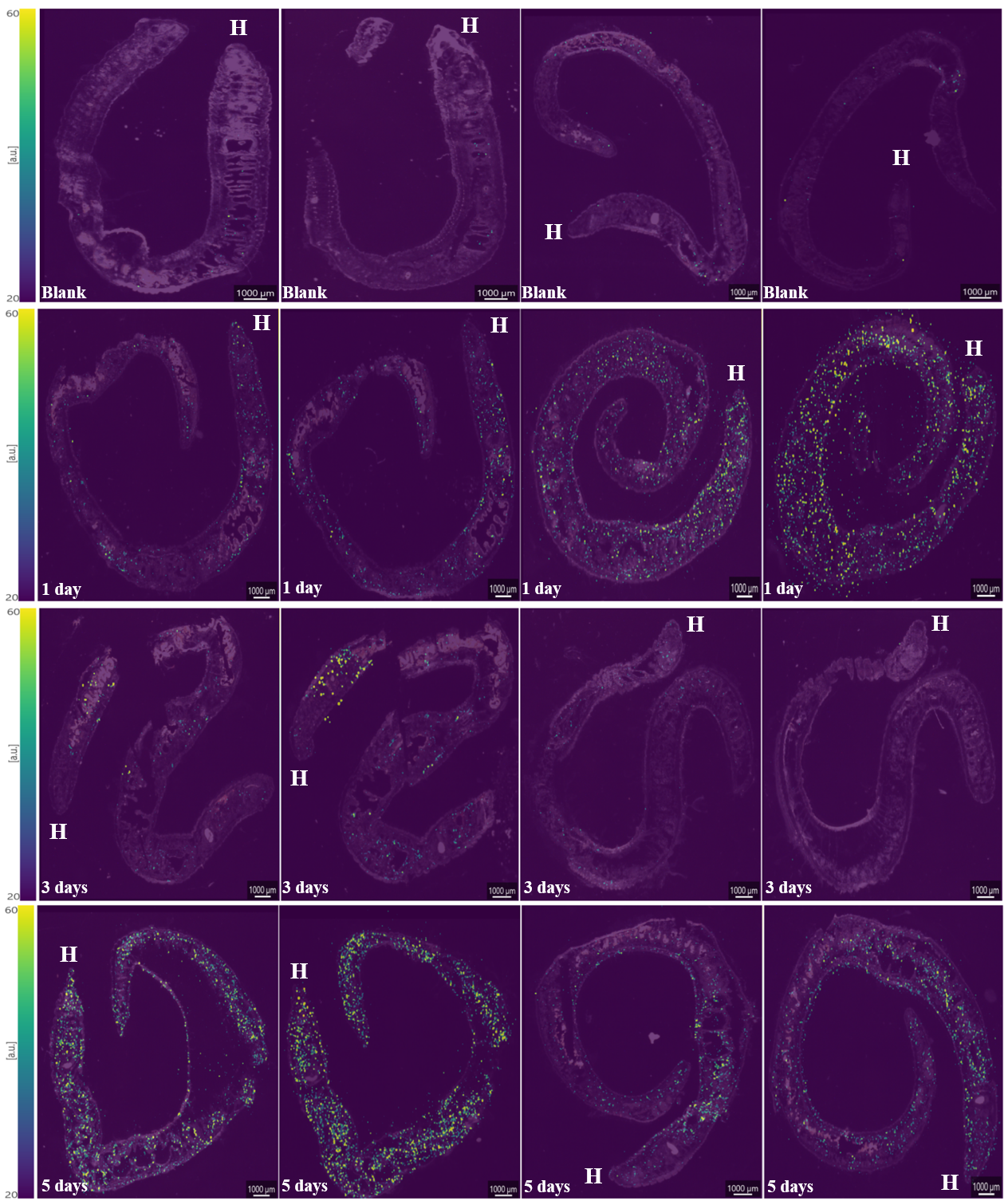
**

**Fig. S5** Spatial distribution of Erythromycin within earthworm tissue. Time of earthworm exposure to contaminated soil is noted in Bottom Left, and 'H' Indicates Earthworm Head Position."

**
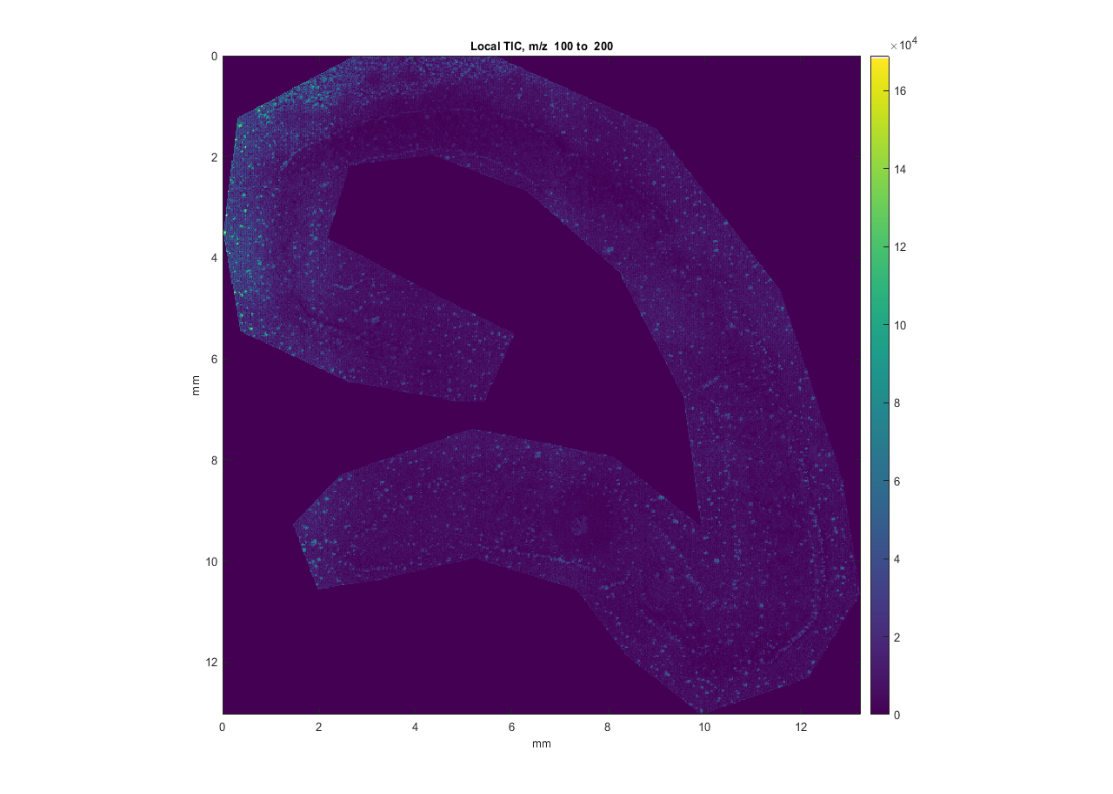

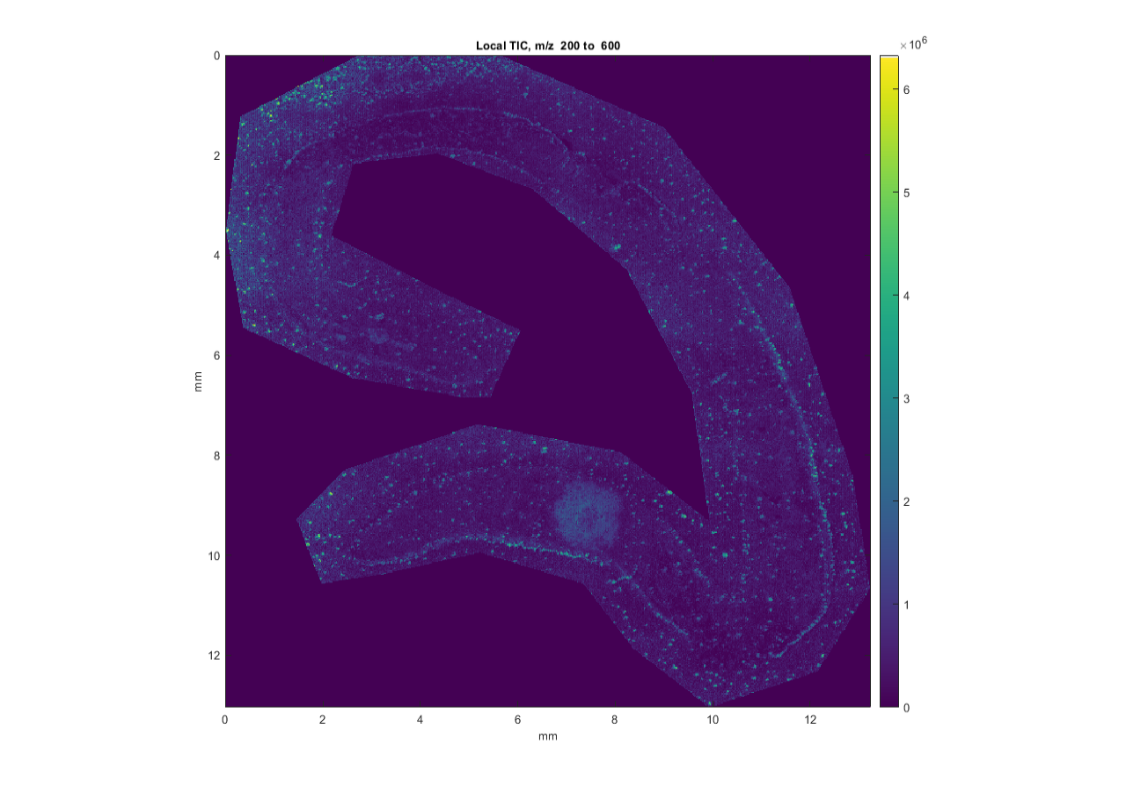
**

**mm**

**Local TIC, m/z 600 to 1,000**

**mm**

**TIC value for each scan**

**mm**

**mm**

**H**

**H**

**Local TIC, m/z 200 to 600**

**Local TIC, m/z 100 to 200**

**
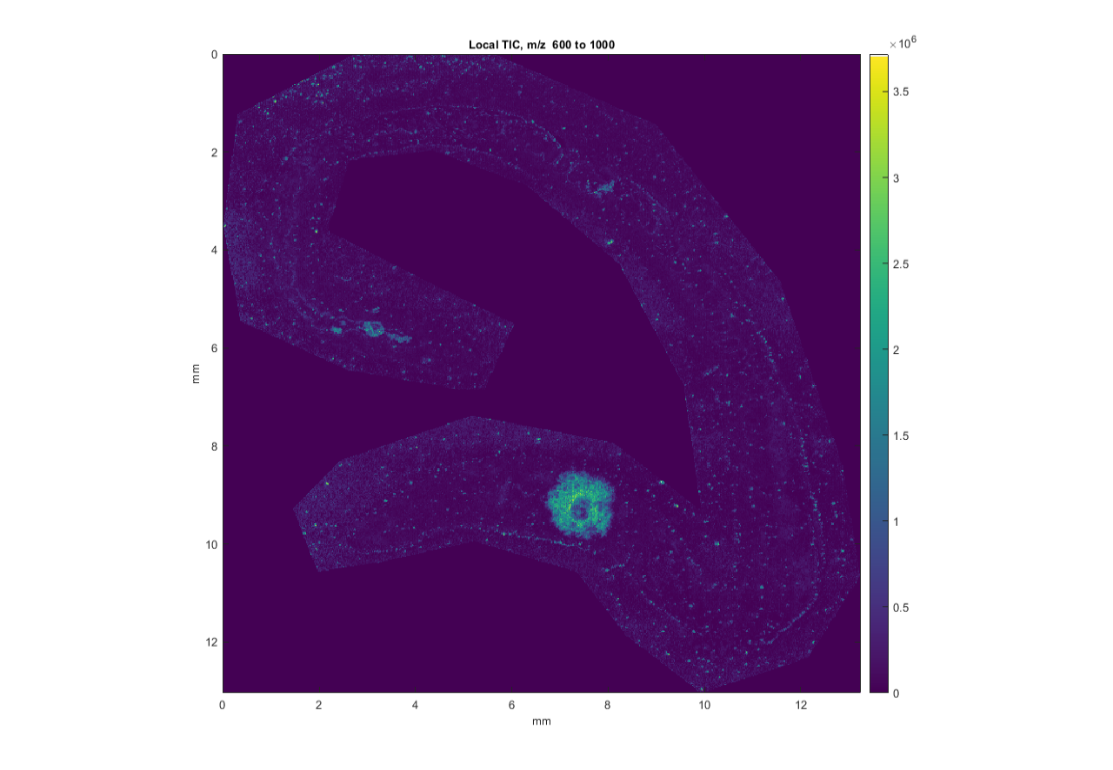

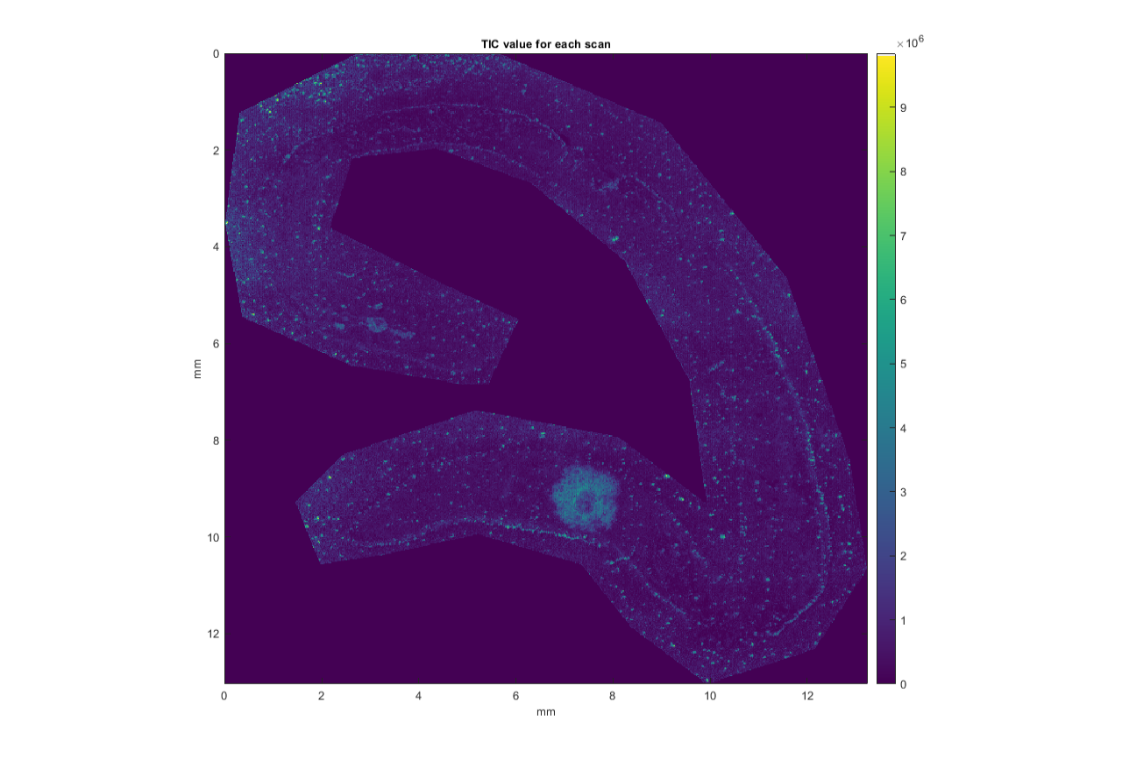
**

**Number of m/z values in each scan (146 569 140)**

**mm**

**mm**

**mm**

**mm**

**H**

**H**

**
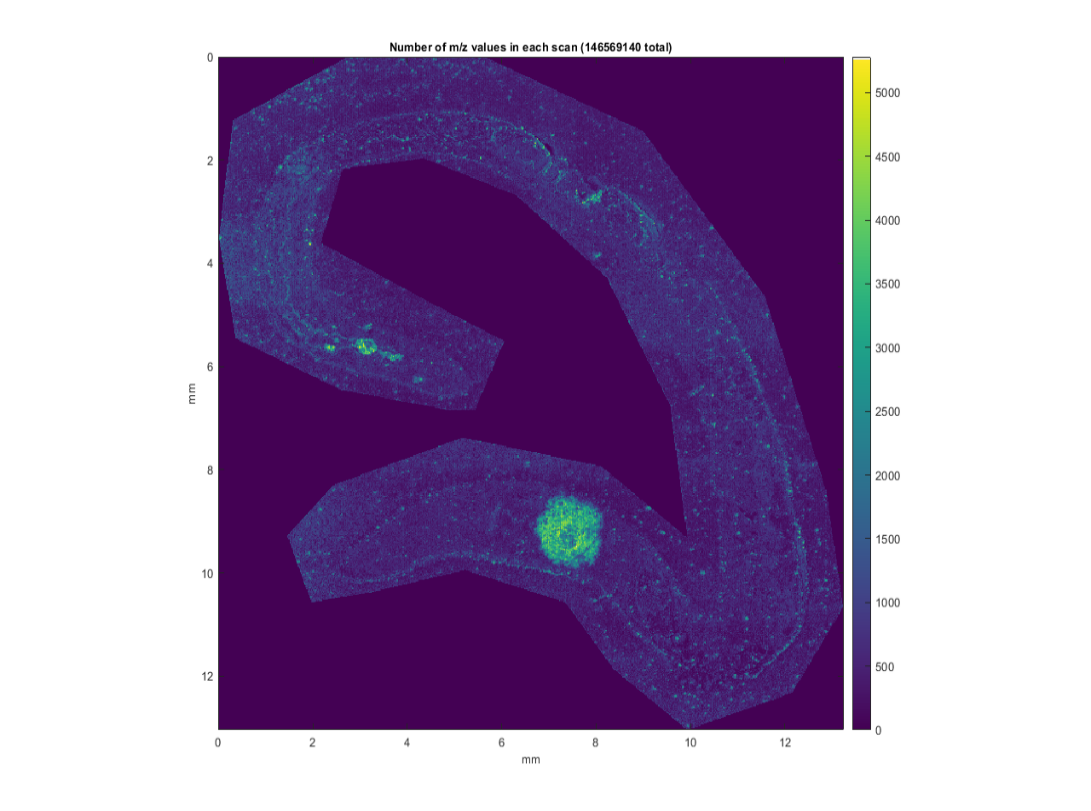
**

**mm**

**mm**

**H**

**Fig. S6** TIC of different mass ranges and Number of Analytes in earthworm tissue, 'H' Indicates Earthworm Head Position."

**REFERENCES**

Holatko J, Brtnicky M, Mustafa A, Kintl A, Skarpa P, Ryant P, Baltazar T, Malicek O, Latal O, Hammerschmiedt T (2023) Effect of Digestate Modified with Amendments on Soil Health and Plant Biomass under Varying Experimental Durations. Materials 16(3):1027. https://doi.org/10.3390/ma16031027

Mravcová L, Amrichová A, Navrkalová J, Hamplová M, Sedlář M, Gargošová HZ, Fučík J (2024) Optimization and validation of multiresidual extraction methods for pharmaceuticals in Soil, Lettuce, and Earthworms. Environmental Science and Pollution Research. https://doi.org/10.1007/s11356-024-33492-7

Xia J, Psychogios N, Young N, Wishart DS (2009) MetaboAnalyst: a web server for metabolomic data analysis and interpretation. Nucleic Acids Res 37(Web Server):W652–W660. https://doi.org/10.1093/nar/gkp356
